# Resolving liquid-to-glass transitions of water under soft nanoconfinement

**DOI:** 10.1038/s41467-026-72955-y

**Published:** 2026-05-08

**Authors:** Patrick Züblin, Eva Zunzunegui-Bru, Livia Salvati Manni, Alice Klapproth, Richard Mole, Nageshwar Rao Yepuri, Syrine Khaled, Guillaume Pierre Laurent, Thierry Azaïs, Serena Rosa Alfarano, Jean-Blaise Brubach, Salvatore Assenza, Francesco Sciortino, Raffaele Mezzenga

**Affiliations:** 1 Department of Health Sciences and Technology, ETH Zürich, Zürich, Switzerland; 2 School of Chemistry and University of Sydney Nano Institute, The University of Sydney, Sydney, Australia; 3 Australian Nuclear Science and Technology Organisation, Clayton, Australia; 4 Australian Nuclear Science and Technology Organisation, Lucas Heights, Australia; 5 Laboratoire de Chimie de la Matière Condensée de Paris (LCMCP), Sorbonne Université, CNRS, Paris, France; 6 Synchrotron SOLEIL, CNRS, Saint-Aubin, France; 7 Departamento de Física Teórica de la Materia Condensada, Universidad Autónoma de Madrid, Madrid, Spain; 8 Condensed Matter Physics Center (IFIMAC), Universidad Autónoma de Madrid, Madrid, Spain; 9 Instituto Nicolás Cabrera, Universidad Autónoma de Madrid, Madrid, Spain; 10 Dipartimento di Fisica, Sapienza Università di Roma, Rome, Italy; 11 Department of Materials, ETH Zürich, Zürich, Switzerland

**Keywords:** Chemical physics, Nanoscale materials, Glasses, Liquid crystals

## Abstract

Water has challenged scientists with numerous anomalies and confounding problems since the very early times. The kinetic glass transition of bulk water is a pristine example, as its location remains mostly inaccessible to experimentalists in an area below about −41 °C, where bulk water spontaneously crystallises. Confining water within nanometre-sized domains is an intriguing approach inspired by the cellular environment in nature to completely prevent crystallisation. Here we probe the existence of liquid-to-glass transitions of water nanoconfined between non-freezing lipidic bilayers and find a peculiar and counterintuitive regime over an extended temperature range, for which a sub-nanometre layer of water remains glassy in between fluid (mobile) walls of lipidic molecules. We provide comprehensive evidence for a slowing down of water dynamics under soft nanoconfinement occurring in the −63 to −20 °C range across six orders of magnitude in time scales from 10^−6^ to 10^−12^ s, and a static glass transition ranging between −74 and −64 °C. These findings provide important insights into the elusive glass transition of water under nanoconfinement, with broader implications for all those areas where water is confined at the nanometre scale below its freezing point.

## Abstract (web summary)

Nanometre-thin water domains confined between fluid, mobile lipid layers exhibit unexpected glassy behaviour, featuring liquid-to-glass transitions at systematically higher temperature than previously reported ranges for water under soft nanoconfinement.

## Introduction

Water is fundamental to all known forms of life and is present on Earth as liquid, solid or vapour. Despite its ubiquitous nature, water presents many physical anomalies, some of which are still unsolved to date^1,2^. In its solid state, water can either form crystalline ices^3,4^ or amorphous solids^5,6^, commonly referred to as glassy water. Although seemingly rare on Earth, the glassy form of water is found in polar stratospheric clouds^7^ and helps psychrophilic microorganisms to survive in cold environments^8^. The glass transition (*T*_g_) of water identifies the kinetic switch from a supercooled liquid to a glass, where water molecules are kinetically arrested in a disordered configuration^9^, unlike in ordered crystalline ice where the solid-state behaviour is associated with long-range structural order. After decades-long history of research, the location of bulk water’s glass transition is still debated^10,11^; calorimetric measurements estimate the *T*_g_ at −137 °C (ref. ^12^). The crux of the problem is that glassy water is notoriously challenging to reach in bulk upon cooling: the most recent experiments locate the homogeneous nucleation temperature around −45 °C (ref. ^13^), below which spontaneous crystallisation of even the purest water occurs under ambient pressure^14–16^ and which is commonly referred to as no-man’s land.

Two of the most established ways to experimentally bypass crystallisation and access glassy water are extremely rapid cooling rates (in the order of 10^5^–10^6 ^K s^−1^)^17,18^ or to confine water in nanometre-sized domains^19,20^. The technique of rapid cooling has contributed to major advances in science such as the invention of cryo-electron microscopy^21^ but it makes the direct observation of *T*_g_ difficult and restricted to experimental settings far from everyday significance. On the other hand, nanoconfinement allows for arbitrarily slow cooling and heating rates while physically preventing water molecules from arranging in a crystalline structure^19,22^, effectively maintaining water in a supercooled dynamic state down to cryogenic temperatures. Additionally, it is of paramount relevance for many biological and technological areas, due to the pervasive role of nanoconfined water in general^23^. Yet, nanoconfinement brings its own challenge: hard confinement, such as silica nanopores^24^ or molecular sieves^18^, introduces configurational constraints in the hydrogen bond network, resulting in a strong dependence of the dynamics of water on the confining media^19^. In contrast, soft confining systems such as micelles, liposomes and bilayers overcome this entropic penalty as they have flexible interfaces, allowing more bulk-like behaviour^25^. The reported *T*_g_ values for water under soft nanometre confinement span a broad range from approximately −73 to −101 °C (ref. ^19^). This variability reflects both the roles of the confining surfaces and the kinetic nature of the glass transition itself, which makes its experimental determination inherently method-dependent^17,26^.

**Fig. 1:**
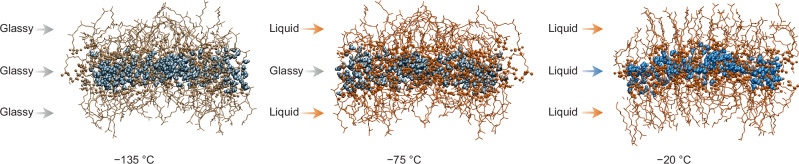
Low-temperature behaviour of water nanoconfined in phytantriol. Schematic illustration of the three dynamical regimes found in water-phytantriol mesophases at representative temperatures based on snapshots from molecular dynamics simulations. The colour saturation in the molecular dynamics snapshots highlights the dynamic state of the water layer (light and dark blue indicating glassy and liquid regimes, respectively) and of the phytantriol molecules (light and dark brown indicating glassy and liquid regimes, respectively). At very low temperatures (−135 °C and below), both the lipid tails and the water domains are in a glassy state. At higher temperatures (−20 °C and above), both components are liquid. An intermediate regime exists (e.g., around −75 °C), where sub-nanometre layers of water are glassy and confined between liquid lipid tails.

Here we tackle this daunting task by moving beyond this classical picture of *T*_g_. We resolve the slowing down of the dynamics of soft nanoconfined water across six orders of magnitude in time scales, from 10^−6^ to 10^−12^ s, by an unprecedented combination of dynamic (quasi-elastic neutron scattering, synchrotron THz absorption spectroscopy and solid-state NMR), static (X-ray scattering) and in silico (atomistic molecular dynamics simulations) methods, applied to self-assembled lipidic mesophases of water and an aliphatic alcohol (phytantriol, see Methods) as a model system for soft nanoconfinement. A typical length scale commonly used to distinguish between the liquid and glassy state is when molecular self-diffusion falls below the nearest-neighbour distance (≈0.28 nm for the water molecule^27^) within the experimental observation time^9^. However, given the extremely vast range of time scales probed here by a multitude of diverse methods, we refrain to point directly and solely to the *T*_g_, and we more generally refer to dynamic arrest on the experimental time scale, accounting for possible observations of both *α*- and *β*-relaxations. We then reach a coherent picture in which dynamic arrest, that is, the marked suppression of relaxation over the time window probed by the experimental techniques employed here, occurs in a temperature range significantly higher than previously reported for soft nanoconfined water. In particular, we identify a temperature regime in which sub-nanometre layers of glassy water are confined between layers of liquid lipidic tails (Fig. 1).

## Results and Discussion

**Fig. 2:**
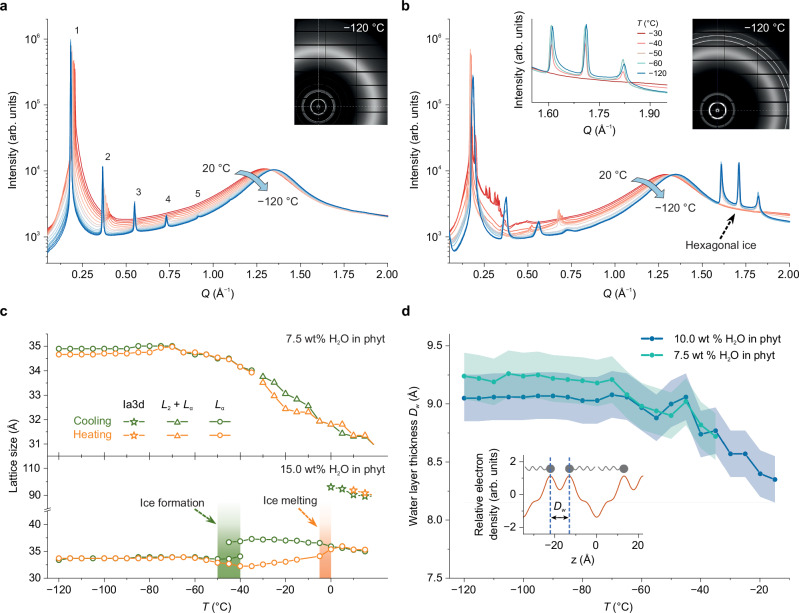
Structural characterisation of non-freezing lipidic mesophase using SAXS/WAXS. Selection of SAXS/WAXS profiles of 7.5 wt% (**a**) and 15.0 wt% H_2_O in phytantriol (**b**) during cooling from 20 to −120 °C. The cooling rate was 1 °C min^−1^. Temperatures are represented by discrete colours in 10 °C increments, ranging from red (20 °C) to dark blue (−120 °C). The first five Bragg reflections (low-*Q* to higher-*Q* order) of the *L*_α_ phase are labelled in **a**. Top-right insets show a 2D-detector image taken at −120 °C. Top-centre inset in **b** shows a zoom of hexagonal ice Bragg reflections. **c**, Lattice size and phase symmetry (Ia3d, *L*_2_ + *L*_α_, *L*_α_) for 7.5 and 15.0 wt% H_2_O in phytantriol as a function of temperature during the cooling and heating cycle derived from Bragg reflections as shown in **a** and **b** and described in “Methods”. **d**, Examples of the water layer thickness (*D*_*w*_) as a function of temperature in the pure lamellar phase (*L*_α_) of 7.5 and 10.0 wt% H_2_O in phytantriol, obtained from the EDP. The error bands represent the uncertainty arising from the instrumental resolution and do not reflect statistical variability from independent measurements. Inset, representative location of the water layer (blue dashed lines) between lipidic headgroups in the EDP. phyt, phytantriol; arb. units, arbitrary units. Source data are provided as a Source Data file.

Small- and wide-angle X-ray scattering (SAXS/WAXS) were used to characterise the low-temperature phase behaviour of phytantriol-water mesophases with different water contents. SAXS/WAXS profiles are a fingerprint of the structural organisation of materials on the Ångstrom to nanometre scale, where the scattering vector *Q* is inversely related to the real-space length scale by 2π/*Q*. Figure 2a shows time-resolved SAXS/WAXS profiles of a lipidic mesophase consisting of 7.5 wt% H_2_O in phytantriol upon cooling from 20 to −120 °C. The absence of characteristic hexagonal ice reflections^28^ at *Q* = 1.61, 1.71 and 1.82 Å^−1^ revealed that H_2_O is not crystalline. We show the non-freezing state of water for 10.0 wt% H_2_O in phytantriol and 8.3 wt% D_2_O in phytantriol in an identical manner in Supplementary Fig. 1a. When the water content is increased to 15.0 wt% (Fig. 2b), H_2_O crystallised between −40 and −50 °C, consistent with a previously determined crystallisation limit of ∼10 wt% to prevent freezing of H_2_O confined in phytantriol-based mesophases^29^.

From the positions of the Bragg reflections in the scattering profiles, we obtained the temperature-evolution of the mesophase symmetries and lattice sizes (Fig. 2c and Supplementary Fig. 2, see Methods for details). Upon cooling 7.5 wt% H_2_O in phytantriol from 20 to −120 °C, the symmetry shifted from a mixed reverse micellar and lamellar (*L*_2_ + *L*_α_) to a highly ordered lamellar phase (*L*_α_) via an order-order transition (OOT). The *L*_α_ phase remained fluid and non-crystalline down to −120 °C due to the non-freezing properties of phytantriol^29^, which is supported by a broad structure factor peak centred at *Q* ∼1.3 Å^−1^ in Fig. 2a. As a control, Supplementary Fig. 1b shows a lamellar crystalline phase formed in a monoolein-based mesophase of identical composition, which rapidly froze between −5 and −10 °C. In the case of phytantriol, the lattice size slightly increased due to tighter lateral packing associated with reduced thermal mobility upon cooling before reaching a constant value of ∼34–35 Å below −60 °C (Fig. 2c, top panel). The phase behaviour was fully reversible upon heating, with no observable thermal hysteresis or metastable phases that would indicate supercooling-related effects. By contrast, the mesophase with 15.0 wt% H_2_O exhibited structural hysteresis due to the formation and melting of ice: upon cooling, the lattice first increased in the gyroid cubic (Ia3d) and lamellar phase before dropping during ice formation (Fig. 2c, bottom panel). The brief coexistence of two lamellar phases at −40 °C and −45 °C suggests that water crystallised only partially outside the lamellar membrane stacks^30^ (Supplementary Information S3). Conversely, the lattice size was fully recovered as the ice melts upon heating. From the electron density profiles (EDPs, see Methods), we obtained a thickness of the confined water layer (*D*_*w*_) of 8–9 Å in the low-temperature regime for non-freezing water contents between 7.5 and 10.0 wt% H_2_O in phytantriol, which are the primary focus in this work (Fig. 2d). We can appreciate a small difference in *D*_*w*_ below about −70 °C which lies within the error. Using SAXS/WAXS, we demonstrated the consistent feature of phytantriol-based mesophases to provide sub-nanometre layers of non-freezing water confined between lipidic bilayers, identifying the static and dynamic regimes of interest in this study.

**Fig. 3:**
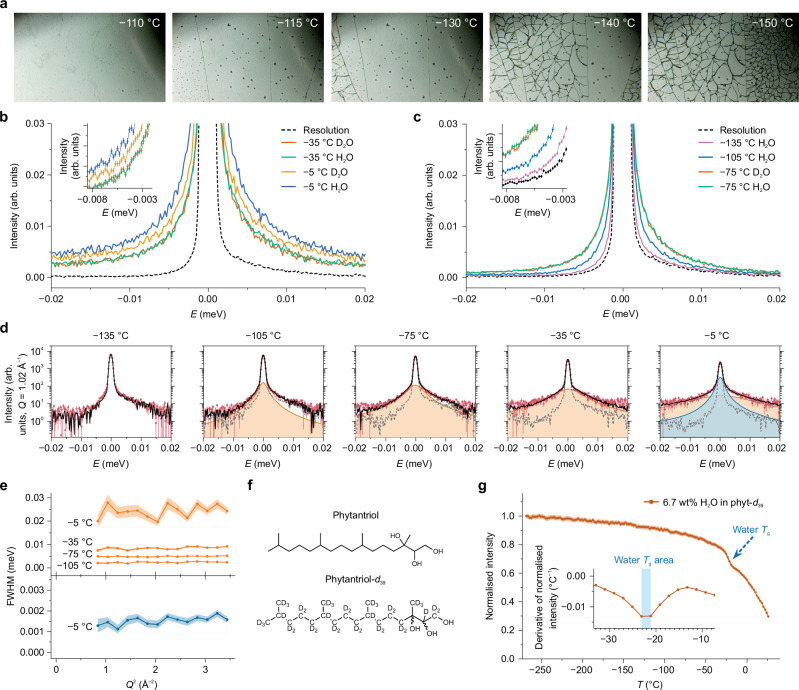
Dynamics from the micro- to the nanometre length scale between − 150 and − 5 °C based on microscopy and neutron scattering. **a** Brightfield microscopy images of crack formation in 7.5 wt% H_2_O in phytantriol at different temperatures. Similar results were observed in three independent experiments on separate samples. The cooling rate was 1 °C min^−1^. Scale bars, 500 μm. **b**, **c** *Q*-integrated QENS spectra of 7.5 wt% H_2_O in phytantriol and 8.3 wt% D_2_O in phytantriol at selected temperatures between −5 and −135 °C. The instrument resolution is represented by the black dashed line. Insets show a zoom of the QENS signal in a selected energy transfer (*E*) range. **d** Fits to the QENS data of 7.5 wt% H_2_O in phytantriol at a fixed *Q*-value of 1.02 Å^−1^ from −135 to −5 °C (see Supplementary Fig. 5 for fits at *Q* = 0.42 and 1.72 Å^−1^). Experimental data are shown in red, the instrument resolution as a grey dashed line and the total fit as a black solid line. The Lorentzian contributions are shown in orange (phytantriol) and blue (water). For **(b**–**d**), error bars represent propagated counting statistics (square root of neutron counts) as mean ± standard deviation and do not reflect variability from independent measurements (*n* = 1). **e** FWHM of the Lorentzians shown in **d** and Supplementary Fig. 5 as a function of *Q*^2^. Error bands denote mean ± standard deviations of the fitted parameters. **f** Chemical structures of hydrogenous phytantriol and deuterated phytantriol-*d*_*39*_, in which all but three non-exchangeable protons in the headgroup are replaced with deuterium. **g** Elastic FWS of 6.7 wt% H_2_O in phytantriol-*d*_39_. The error band denotes the mean ± standard deviation of the neutron counts and do not reflect variability from independent measurements (*n* = 1). Inset, first derivative of the normalised intensity. arb. units, arbitrary units. Source data are provided as a Source Data file.

Upon cooling a mesophase of 7.5 wt% H_2_O in phytantriol to cryogenic temperatures, cracks formed on a macro- and microscopic level starting at −115 °C, revealing a brittle glassy state of the entire bulk mesophase (Fig. 3a, Supplementary Fig. 4). This static visual observation allowed us to identify a temperature range of interest for the dynamics study.

Quasi-elastic neutron scattering (QENS) is a powerful tool to selectively capture the dynamics of hydrogen atoms. Via isotopic contrast variation, it is possible to isolate the dynamics of single components, as the incoherent scattering cross-section of deuterium is nearly 40 times smaller than that of hydrogen^31^. We used the backscattering spectrometer Emu to probe diffusive motions of hydrogenous components in a time window of ∼130 ps to ∼4.5 ns, by analysing the quasi-elastic broadening around the elastic line (*E* = 0 meV). Figures 3b and 3c show QENS spectra of 7.5 wt% H_2_O in phytantriol and its molar equivalent of 8.3 wt% D_2_O in phytantriol at selected temperatures between −5 and −135 °C, integrated over a *Q*-range from 0.42 to 1.72 Å^−1^. At −5 °C, the QENS spectrum of the mesophase with H_2_O was significantly broader than that of the mesophase containing D_2_O (Fig. 3b), consistent with an enhanced mobility of H_2_O molecules on a nanosecond time scale. By contrast, at −35 °C and −75 °C the QENS spectra of the mesophases with H_2_O and D_2_O overlapped within experimental uncertainty (green and red curves in Fig. 3b, c); this observation is only compatible with kinetically arrested molecular motion of water, thus revealing that water was glassy on a nanosecond time scale at −35 °C and colder temperatures, where any QENS broadening and thus dynamics originated exclusively from phytantriol.

The dynamics encoded in the quasi-elastic broadening of the elastic peak are typically analysed by fitting a Lorentzian curve for each type of motion^32^. While there was no signal broadening and therefore no dynamics within the probed time window at −135 °C, we observed the onset of a distinct lipidic motion at −105 °C on the nanosecond time scale, based on a single Lorentzian contribution (Fig. 3d and Supplementary Fig. 5). Additionally, we identified a boson peak in the inelastic region at around −2.2 meV and −135 °C on the time-of-flight spectrometer Pelican probing the sample of 7.5 wt% H_2_O in phytantriol on a picosecond time scale (Supplementary Fig. 6). The boson peak is recognized as a hallmark of glassy materials and arises from an excess in the vibrational density of states as compared to the Debye behaviour^33,34^. We therefore conclude that phytantriol hydrated with 7.5 wt% H_2_O undergoes a liquid-to-glass transition between −105 and −135 °C on a nanosecond time scale. Upon increasing the temperature from −105 °C, the Lorentzian contribution from phytantriol broadened, indicating that the motion became faster in the nanosecond time domain (Fig. 3d, Supplementary Fig. 5). At −5 °C, the data are best described by fitting a second, narrow Lorentzian, which can be attributed to the simultaneous water mobility. The full-width at half-maximum (FWHM) of the Lorentzian as a function of *Q* provides information on the time and length scales of the dynamics^32^. The *Q*-independent linewidths indicate localised dynamics for phytantriol and water (Fig. 3e), with no evidence for Fickian or Singwi–Sjölander jump diffusion^35^ down to the maximum probed length scale of ∼15 Å (*Q* = 0.42 Å^−1^).

An elastic fixed window scan (FWS) of 6.7 wt% H_2_O in phytantriol-*d*_39_ enabled us to locate the liquid-to-glass transition of soft confined water between −23 and −21 °C (Fig. 3f,g). This observation is further supported by a pronounced increase in the apparent mean-squared displacement extracted from the *Q*-dependence of the elastic intensity at low *Q* (ref. ^32^) (Supplementary Fig. 7). Deuterated phytantriol-*d*_39_ allows to increase the relative incoherent neutron scattering contribution from H_2_O from around 7% to 42% (Supplementary Table 1) when comparing molar-equivalent mesophases of 7.5 wt% H_2_O in hydrogenous phytantriol with 6.7 wt% H_2_O in phytantriol-*d*_39_. At the same time, 6.7 wt% H_2_O in phytantriol-*d*_39_ exhibits a low-temperature phase behaviour similar to that of hydrogenous phytantriol^36^, as confirmed by SAXS/WAXS (Supplementary Fig. 1a). Based on a comprehensive analysis of neutron scattering experiments, we thus identified an elevated temperature range for the liquid-to-glass transition of sub-nanometric soft confined water between −35 °C (lower limit determined by QENS analysis) and −21 °C (upper limit given by FWS) on a nanosecond time scale.

**Fig. 4:**
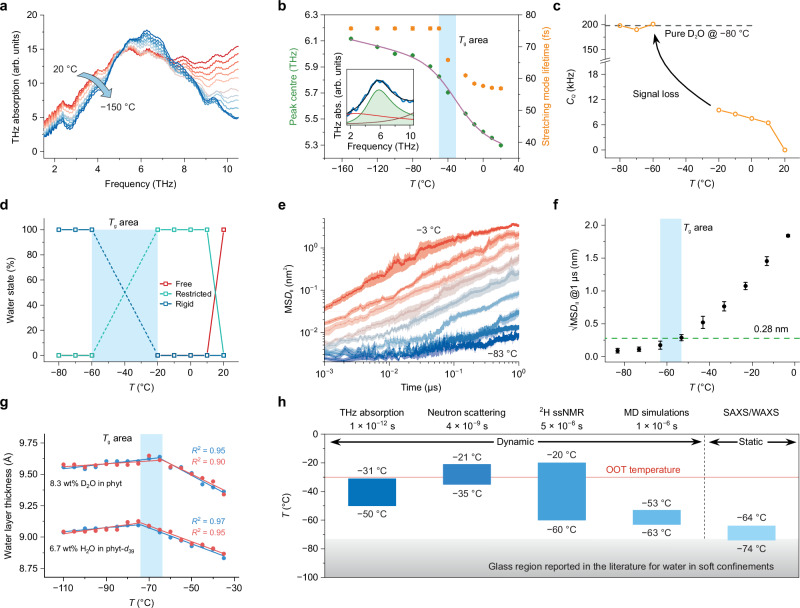
Locating the liquid-to-glass transitions of water under lipidic nanoconfinement. **a** Selection of isolated water THz absorption spectra of 10.0 wt% H_2_O in phytantriol upon cooling from 20 to −150 °C. The temperature rate was 1 °C min^−1^. Temperatures are represented by discrete colours in 10 °C increments, ranging from red (20 °C) to dark blue (−150 °C). **b** Intermolecular stretching mode frequency of H_2_O (green dots, left *y* axis) with a sigmoidal curve fit (purple line, see Supplementary Table 6 for fit parameters) and stretching mode lifetime (orange dots, right *y* axis) as a function of temperature in 10.0 wt% H_2_O in phytantriol. Data are presented as mean ± standard deviation of three scan repetitions. Error bars are smaller than dots. Inset, example of the spectral fit showing the stretching mode of water (green), Debye relaxation (red), libration mode (brown), experimental data (blue) and the total fit (black). **c**, Thermal evolution of the ^2^H NMR quadrupolar coupling constant (*C*_*Q*_) in 8.3 wt% D_2_O in phytantriol. **d**, Water state percentages in 8.3 wt% D_2_O in phytantriol derived from the *C*_*Q*_ value analysis in **c**. **e**, MS*D*_∥_ of H_2_O in a system of 10.0 wt% H_2_O in phytantriol obtained from atomistic molecular dynamics simulations at temperatures ranging from −3 to −83 °C in 10 °C increments. Temperatures are colour-coded from red (−3 °C) to dark blue (−83 °C). **f**, Average displacement of water molecules (√MS*D*_∥_) at 1 μs. The dashed green line indicates the nearest-neighbour distance of water molecules. Data in **e** and **f** are presented as mean ± standard deviation from five independent runs (*n* = 5). **g**, Water layer thickness as a function of temperature in two mesophases, obtained from EDPs as described in Methods. The *T*_g_ range is defined by the breakpoints of the piecewise linear regression model (see Supplementary Fig. 14 for fit parameters), applied to the linear regimes of the cooling (blue) and heating (red) cycles. The temperature rate was 1 °C min^−1^. Curves are offset and rescaled vertically for clarity. **h** Overview of the dynamic arrest temperature range (in blue) determined in this work by various techniques. The *T*_g_ range of water in different soft confinements reported in the literature^19,29^ is indicated by the grey area. The order-order transition (OOT) temperature at −30 °C, below which the system exists as a single phase, is represented as a solid red line. arb.units, arbitrary units. Source data are provided as a Source Data file.

We then probed the liquid-to-glass transitions of the soft nanoconfined water from the picosecond to microsecond time scale using complementary THz absorption spectroscopy, ^2^H solid-state NMR, and molecular dynamics simulations. THz absorption spectroscopy was used to resolve the picosecond time scale, enabling the excitation of the hindered translation vibrational mode of water, which occurs at approximately 6 THz and is also referred to as the intermolecular stretching mode in this frequency regime. Upon cooling a mesophase of 10.0 wt% H_2_O in phytantriol from 20 to −150 °C, the spectra underwent a blueshift (Fig. 4a), which is related to an increased rigidity of the hydrogen bond network. This shift is quantitatively analysed using a modified harmonic oscillator model that effectively captures the three key vibrational processes in the low-frequency regime (see Methods, Supplementary Figs. 8–11 and Supplementary Tables 2–5), with particular emphasis on the stretching mode^37^ (Fig. 4b, inset). Its centre frequency exhibited a sigmoidal temperature dependence (green dots in Fig. 4b). Such a dynamical cross-over is well-established for supercooled confined water and is frequently described as the transition from fragile non-Arrhenius to strong Arrhenius behaviour^10,19,38^, characteristic of network-forming liquids close to their glass transition. Note that the roughly linear blueshift observed below about –50 °C is akin to the behaviour seen in crystalline ice (Supplementary Fig. 12), corroborating that water has reached a kinetically arrested state attributable to glassy water. On the other hand, the width of the stretching mode is inversely proportional to its lifetime^39^, although it may include contributions from inhomogeneous broadening mechanisms. The lifetime initially increased monotonically upon cooling (Fig. 4b, orange dots) and, below about –20 °C, increased abruptly before reaching a plateau at –50 °C. This plateauing suggests that, within the rigid and kinetically arrested hydrogen bond network, water molecules are no longer able to further slow their vibrational dynamics. Combining these findings, we locate a range from −31 to −50 °C for the dynamic arrest of water on the THz time scale (1 × 10^−12^ s), bordered by the inflection point of the sigmoid-like curve (see Methods) and the plateauing of the stretching mode lifetime.

^2^H ssNMR provides information on the reorientation of water molecules, while the quadrupolar coupling constant (*C*_*Q*_) reflects the dynamics of the deuterium nuclei within the molecule. The *C*_*Q*_ initially increased from 0 to 9.5 kHz upon cooling the mesophase with 8.3 wt% D_2_O in phytantriol from 20 to −20 °C, which reveals a slowing of the D_2_O molecules from a free bulk state (fast isotropic reorientation; *C*_*Q*_ = 0 kHz) to an anisotropic restricted liquid-like state (*C*_*Q*_ of a few kHz) (Fig. 4c,d), consistent with previous studies on water in lipidic bilayer systems^22,40–42^. The sudden loss of the NMR signal between −30 and −50 °C originates from a transition state of supercooled D_2_O. In this state, dynamics diminish to an intermediate frequency regime, where fast transverse *T*_2_ relaxation prevents signal acquisition^43^ (Supplementary Information S13). At −60 °C, the signal was recovered and the *C*_*Q*_-value converged to that of crystalline D_2_O (*C*_*Q*_ = 198 kHz, horizontal grey dashed line in Fig. 4c), which signifies that D_2_O molecules reach the slow motional limit and can be considered kinetically arrested from the NMR point-of-view (Fig. 4d). Based on this evidence, we conclude that dynamic arrest of confined D_2_O occurs between −20 and −60 °C on the NMR time scale (∼5 × 10^−6^ s). We note in passing that in bulk the calorimetric glass transition of D_2_O is reportedly 10 ± 2 K above that of H_2_O due to isotope effects^44^.

We then performed atomistic molecular dynamics simulations of 10.0 wt% H_2_O in phytantriol to investigate the translational dynamics of water within the system. Specifically, we calculated the mean-squared displacement in the direction parallel to the midplane of the membrane (MS*D*_‖_) from −3 to −83 °C, reaching simulation times of 1 μs (Fig. 4e). The maximum MS*D*_‖_ values decreased upon cooling, reflecting the reduced mobility of water characteristic of supercooled dynamics^45,46^. In particular, the average displacement of water molecules at 1 μs decreased steadily with temperature (Fig. 4f). At −63 °C, the average displacement drops below the nearest-neighbour distance of water molecules ( ≈ 0.28 nm) (ref. ^27^) on the microsecond time scale, indicating the kinetically arrested state of glassy water^9,47,48^. Therefore, we estimate a liquid-to-glass transition of sub-nanometric confined water between −53 and −63 °C on a simulated microsecond time scale.

All the methods discussed so far probed specific time scales. By contrast, structural analysis allows a possible decoupling from rate-dependent measurements. We observed that the temperature dependence of the water layer thickness derived from the EDPs abruptly changed across different samples during both cooling and heating cycles at about −70 °C (Fig. 4g, see also Fig. 2d). Given the 2D symmetry, changes in thickness directly reflect changes in volume (i.e. density); hence, we can locate the *T*_g_ by examining the change in slope, i.e. the first derivative evolution of this value with respect to temperature. Since phytantriol shows diffusive mobility on a nanosecond time scale as low as −105 °C and is therefore fluid in this range, the apparent changes in the temperature dependence can solely be ascribed to water. These measurements of volumetric changes further support the presence of a static liquid-to-glass transition between −64 and −74 °C.

Our values for the liquid-to-glass transitions of water under soft lipidic nanoconfinement are found systematically above the previously reported range for the *T*_g_ of water in soft nanoconfinement (Fig. 4h), including earlier values reported by some of us for the same system based on differential scanning calorimetry and broadband dielectric spectroscopy^29^. A conventional framework to explain the dynamics of glass-forming liquids is provided by a topographic energy landscape, which maps the energetic valleys and barriers dictating how a system explores its possible molecular arrangements^26,49,50^. Clearly, this energy landscape varies when water is confined. Additionally, whether water shows (true) *α*-relaxation when under confinement still remains a controversial point in the community^19^. Even when all the above are considered, the glassy sub-nanometre layers of confined water found in our system are surprising when compared to other glassy systems. In other amorphous solids, such as polymers, there is a classic size-depression of the *T*_g_ with film thickness, which is already noticeable at just a few nanometres^51^. Here, we report the observation of a glassy layer of water as thin as 0.8–0.9 nm (∼3 molecules of water) confined between liquid lipidic walls. This suggests that the relaxation processes of water are decoupled from those of the phytantriol hydrophobic tails, which, based on the combined macroscopic behaviour of the mesophase (Fig. 3a) and neutron scattering (Fig. 3d), exhibit signatures of glassy behaviour only at temperatures below −105 °C. In support of this picture, solvation effects can be deemed as a secondary effect here, as glycerol–water mixtures with molar compositions equivalent to our system (71–77 wt% glycerol in H_2_O) exhibit a *T*_g_ below −80 °C (ref. ^52^). Our results indicate that, as expected, the exact location of the liquid-to-glass transition depends on the dynamical time scale probed by the measurement technique. We observe that all glassy regimes identified here remain above the previously reported range for soft nanoconfined water, while techniques probing comparable time scales (MD simulations and ^2^H ssNMR) yield overlapping temperature ranges. We find a narrow window between −31 and −35 °C that is compatible with a dynamic glass transition of water probed across six orders of magnitude in observation time scales by THz absorption spectroscopy, neutron scattering, and ^2^H ssNMR. Importantly, all three experimental dynamical techniques reveal a single dominant relaxation process for water within the identified *T*_g_ range, which extends even above the OOT temperature ( ≥ −30 °C), inferring that water dynamics are not governed by changes in the confining symmetry, consistent with our earlier experimental, numerical and theoretical findings^53^. Finally, static techniques such as SAXS yield a relatively lower temperature range of the liquid-to-glass transition (yet above the previously reported range for the *T*_g_ of soft nanoconfined water), as it should be expected for infinitely slow measurements of the *T*_g_.

Despite the wide range of techniques used and time scales probed, a coherent picture emerges from our work. In view of these results, Franks’^54^ statement that water is among the most studied yet least understood liquids remains as relevant today as it was then. More generally and beyond the purely fundamental aspects, our findings bear immediate and broader relevance to all physical phenomena involving cryogenic nanoconfined water, such as in the cryopreservation of biological material and deep freezing of food, to name only a couple of examples.

## Methods

### Deuterated phytantriol-*d*_39_

Phytantriol-*d*_39_ was synthesised from phytanic acid as described in ref. ^36^, yielding 96.5 ± 2% deuteration at all non-exchangeable proton sites as determined by mass spectrometry (Supplementary Figs. 15 and 16).

### Mesophase preparation

Phytantriol is an amphiphilic aliphatic alcohol that self-assembles with water into well-defined lyotropic mesophases^55^, analogous to those formed by lipid-water systems. Although phytantriol is not a lipid in the strict chemical sense, its molecular architecture and phase behaviour closely resemble those of lipid-based amphiphiles. The term ‘lipidic mesophase’ is used to describe these lipid-like self-assembled nanostructures. Lipidic mesophase samples were prepared by weighing hydrogenous phytantriol or phytantriol-*d*_39_ and H_2_O or D_2_O in the desired weight ratio in Pyrex tubes. The mixtures were gently heated until an isotropic fluid was obtained; then vortexed and centrifuged at 2000 *g* for 10 min. The mesophases were equilibrated for at least 24 h at 25 °C prior to measurements. Mesophases involving deuteration (8.3 wt% D_2_O in hydrogenous phytantriol and 6.7 wt% H_2_O in phytantriol-*d*_39_) were prepared with hydration levels yielding the same molar water-to-phytantriol ratio (and thus molar composition) as in 7.5 wt% H_2_O in hydrogenous phytantriol.

### SAXS/WAXS measurements

The experiments were performed at the SAXS/WAXS beamline of the Australian Synchrotron, Clayton (Australia). The photon energy was set to 12 keV (*λ* = 1.03 Å), resulting in a high X-ray flux ideal for soft condensed matter systems. The focal beam size was 25 × 250 µm, and data were recorded on a Pilatus 2 M detector, located 600 mm from the sample. With this setup, the accessible *Q*-range was from 0.02 to 2.06 Å^−1^, calibrated using silver behenate. The temperature was controlled using a Linkam Small Area Stage (HFS600) connected to an LNP96 liquid nitrogen pump and confirmed by an independent thermocouple. The sample volume was about 50 mm^3^. Before each experimental run, the samples were equilibrated at 25 °C for 30 min. The sample stage was purged with nitrogen to prevent ice formation inside the stage environment due to ambient humidity. Then, the samples were cooled at 1 °C min^−1^ to −120 °C (cooling cycle), left at −120 °C for 30 min, and the temperature was subsequently increased at a rate of 1 °C min^−1^ to 25 °C (heating cycle). SAXS/WAXS scattering profiles were acquired in 5 min steps throughout the experimental run and the scattered intensity was collected for 1 s. The data were processed using the in-house developed ScatterBrain software version 2.82 (ref. ^56^). The mesophase symmetries are identified by the relative positions of the Bragg peaks in *Q*, which follow the characteristic pattern *n* = √6:√8:√14:√16:√20:√22 for the gyroid cubic Ia3d phase and *n* = 1:2:3… for the lamellar (*L*_α_) phase. The reverse micellar (*L*_2_) phase is identified by a single broad peak. The lattice size (*a*) is then derived using Bragg’s law:

1 $$\,a=\,\frac{2\pi }{Q}n$$

The electron density profiles were constructed via Fourier analysis from the SAXS/WAXS data considering the first 4 peaks of the lamellar (*L*_α_) phase as described in refs. ^57,58^. The thickness of the water layers was determined by the peak-to-peak distance of Gaussian fits to the electron density profile, as shown in the inset of Fig. 2d.

### Low-temperature microscopy

Brightfield microscopy images were acquired on a Zeiss Axio Imager Z2 with an AxioCam MRc and using a Linkam temperature control stage (LTS350) connected to an LNP96 liquid nitrogen pump. Approximately 200 mg of the sample was placed on a standard microscopy slide and covered with a cover glass. The sample was cooled at a rate of 1 °C min^−1,^ and the stage was purged with nitrogen to prevent ice formation due to ambient humidity.

### Neutron scattering

QENS measurements were conducted on the backscattering spectrometer Emu^59^ and on the time-of-flight spectrometer Pelican^60^ at the Australian Centre for Neutron Scattering in Lucas Heights (Australia). Backscattering QENS and elastic FWS experiments were performed on the Emu spectrometer with an energy resolution of 1.1 μeV using an incident wavelength of 6.27 Å, resulting in an accessible *Q*-range of 0.32–1.72 Å^−1^ and a time window of ∼0.13–4.5 ns. Elastic FWS were recorded during a temperature ramp from −269 to 27 °C at a rate of 0.5 °C min^−1^, with measurements taken at 2 °C intervals. Time-of-flight experiments used 6 Å neutrons and an energy resolution of 65 μeV, resulting in an accessible *Q*-range of 0.32–1.72 Å^−1^ and a time window of ∼1–20 ps. The samples were loaded in annular cans with 0.2 mm gap size, corresponding to an estimated transmission of approximately 90%. Data were normalised and calibrated using a vanadium standard sample. Scattering from an empty sample, measured under the same instrumental configuration and temperature conditions as the samples, was subtracted as background. For QENS analysis of the backscattering experiments, the lowest practical *Q*-value used in the analysis was 0.42 Å^−1^, owing to insufficient counting statistics in the lowest-*Q* bin at the measured temperatures. Data were binned in increments of 0.1 Å^−1^ in a *Q*-range of 0.42–1.72 Å^−1^ and fitted with a Lorentzian for each quasi-elastic contribution. Data were numerically convoluted with the instrument resolution^32^, leading to small point-to-point fluctuations in the fitted curves that do not affect the extracted linewidths. The Emu backscattering spectrometer exhibits a known resolution asymmetry, which is reflected in the raw spectra and accounted for in the convolution. No additional background component was required in the best overall fit. Data reduction and analysis were performed using the Mantid software package v.6.10.0 (ref. ^61^).

The apparent mean-squared displacement (MSD) was obtained from the *Q*-dependence of the elastic FWS data using a Gaussian approximation:

2 $${{\mathrm{ln}}}{I}_{{el}}\,(Q)=-\frac{{Q}^{2}}{3}\left\langle {u}^{2}\right\rangle$$

where ${I}_{{el}}$ is the elastic intensity and $\left\langle {u}^{2}\right\rangle$ the MSD. The *Q*-range considered for the analysis was 0.32–0.92 Å^−1^ and 150 temperature points between −269 and 27 °C were evaluated. The uncertainty of the MSD was derived from the standard error of the fitted slope.

The reduced vibrational density of states ($g(\omega )/{\omega }^{2}$) was derived from the time-of-flight data using the ComputeIncoherentDOS algorithm implemented in Mantid. In brief, $S(Q,\omega )$ is transformed to the generalised density of states within the incoherent approximation using:

3 $$\,g(Q,\omega )=\frac{\hslash \omega }{{Q}^{2}}S(Q,\omega )\left(1-{e}^{\frac{-\hslash \omega }{{kT}}}\right)$$

where the scattering function $S(Q,\omega )$ is corrected for temperature-dependent phonon populations using the Bose factor at energy $E={\hslash }\omega$. We then integrate g$(Q,\omega )$ over the measured *Q*-range for each energy. Finally, the spectrum is weighted by ${\omega }^{2}$, which yields $g(\omega )/{\omega }^{2}$ to emphasise the boson peak.

### THz absorption spectroscopy

THz absorption experiments were performed at the AILES beamline of the SOLEIL synchrotron (Gif-sur-Yvette, France). The spectra were recorded in transmission mode using a Bruker IFS 125 Fourier transform spectrometer. A 6 μm multilayered beamsplitter and a 4.2 K Si bolometer detector were employed to cover the THz spectral range from 0.5 to 16 THz. Each spectrum was recorded at a resolution of 2 cm^−1^ acquired from 64 scans, and averaged over three repetitions. Samples were placed in a copper cell between two diamond windows connected to a pulsed tube cryostat (Cryomech), allowing to record spectra from 20 to −150 °C. Samples were cooled at 1 °C min^−1^ and equilibrated for 10 min prior to each measurement. The sample thickness was 70 μm (10.0 wt% H_2_O in phytantriol) and 60 μm (15.0 wt% H_2_O in phytantriol). The contribution from pure phytantriol was subtracted from the spectra before fitting.

The temperature-dependent absorption coefficient ($\alpha (\nu,T)$) of the sample is calculated with the Beer-Lambert law^62^:

4 $$\,\alpha \left(\nu,T\right)=\frac{1}{d}\log \left(\frac{{I}_{0}\left(\nu,T\right)}{I(\nu,T)}\right)$$

where $d$ = 70 and 60 µm is the sample thickness in 10.0 and 15.0 wt% H_2_O in phytantriol, respectively, ${I}_{0}$ is the incident intensity, $I$ is the transmitted intensity and $\nu$ is the frequency of the incident light, which is between 50 and 350 cm^−1^.

For all samples, we subtracted the absorption spectrum of phytantriol (${\alpha }_{{phy}}(\nu,T)$), weighed by its volume fraction (${\phi }_{{phy}}$), to isolate the contribution of water to the overall spectrum:

5 $$\,\Delta \alpha \left(\nu,T\right)=\alpha \left(\nu,T\right)-{\phi }_{{phy}}{\alpha }_{{phy}}\left(\nu,T\right)$$

This spectrum is modelled with^37,53^:

6 $$\,\Delta \alpha \left(\nu,T\right)={\sum }_{i=1}^{N}{{{\mathscr{L}}}}_{i}(\nu,T)$$

where ${{{\mathscr{L}}}}_{i}\left(\nu \right)$ is a slightly modified form of the damped harmonic oscillator^63^, which models the intermolecular modes of water as:

7 $${\,{{\mathscr{L}}}}_{i}\left(\nu,T\right)=\frac{{a}_{i}{w}_{0,i}^{2}{\nu }^{2}}{4{\pi }^{3}\left({\left({\nu }_{0,i}^{2}-{\nu }^{2}\right)}^{2}+{\left(\frac{{\omega }_{0,i}}{2\pi }\right)}^{2}{\nu }^{2}\right)\,}$$

where ${a}_{i},\,{\nu }_{0,i},\,{\omega }_{0,i}$ are the amplitude, centre frequency, and width of the intermolecular modes of water, respectively.

Specifically, phytantriol-water samples with 10.0 and 15.0 wt% H_2_O (the latter only above −40 °C) exhibit intermolecular modes of stretching (∼5.4 THz), libration (∼10.5 THz), and Debye relaxation (∼2.4 THz), corresponding to hindered translational, rotational, and bending OH$\cdots$H motions of water molecules^37,64^. The low frequency (LF) is ascribed to the Debye relaxation mode, as established by dielectric relaxation spectroscopy in resonances^65^ (see Supplementary Tables 2,3 and Supplementary Figs. 8–11 for the complete set of data). In contrast, for the sample of 15.0 wt% H_2_O at and below −40 °C where hexagonal ice (Ih) is observed, the intermolecular modes include two stretching modes (centred around 190–200 cm^−1^ or 5.7–6.0 THz) and an additional mode in the 120–165 cm^−1^ (3.6–4.95 THz) range, which is attributed to weakly hydrogen-bonded or the proton disordered water associated with ice Ih^64^ (see Supplementary Tables 4,5 and Supplementary Figs. 10 and 11 for the complete set of data). The stretching mode’s centre frequencies ($\nu (T)$) for 10.0 wt% H_2_O in phytantriol (Fig. 4b) are fitted using a sigmoid plus a linear term:

8 $$\,\nu \left(T\right)={aT}+c+\frac{\left(b-a\right)}{1+{e}^{-\frac{T-{x}_{0}}{{c}_{0}}}}$$

where ${a,{b},{c},{x}}_{0}$ and ${c}_{0}$ are the fitting parameters, with ${x}_{0}$ being the inflection point of the sigmoid and ${c}_{0}$ controlling the width of its steep region. The fitting parameters of the sigmoidal fit are presented in Supplementary Table 6.

### ^2^H solid-state NMR measurements

^2^H ssNMR measurements were conducted on a sample containing 8.3 wt% D_2_O in phytantriol. A 4 mm zirconia rotor was filled using a spatula after gently stirring the sample with a needle to remove air bubbles. The sample was then allowed to equilibrate at room temperature for 60 min. Subsequently, the temperature was stepwise decreased from 20 to −90 °C at a rate of 1 °C min^−1^, with measurements taken at 10 °C intervals. At each temperature, the sample was equilibrated for 20 min prior to acquisition. The sample was cooled by a cold bearing gas of N_2_ that passed through a liquid nitrogen Dewar, and the desired temperature was reached by varying the bearing gas pressure. Solid-state ^2^H NMR spectra were acquired on a 7 T spectrometer (Bruker) at a ^2^H Larmor frequency of 46 MHz, using a 4 mm probe in static mode. A solid-echo sequence was applied with 90° pulses widths of 3.3 µs, an interpulse delay of 30 µs, a recycle delay of 45 s and 80 scans. The spectral width was set to 1 MHz. The proportions of water states were deduced from the decomposition of the ^2^H spectra using pure quadrupolar line shapes. The decomposition and determination of the quadrupolar parameters (*C*_*Q*_ and *η*_*Q*_) were performed using the ssNake program^66^.

### Atomistic molecular dynamics simulations

All molecular dynamics (MD) simulations were performed using GROMACS v.2022.4 (ref. ^67^). Water molecules were modelled using the TIP4P/Ice model^68^. Phytantriol molecules were simulated using the GROMOS 54A7 force field combined with in-house parametrisation for the lipid headgroups to ensure accurate characterisation of hydroxyl group-water interactions, as described in ref. ^53^. Phytantriol coordinates were obtained from the automated topology builder^69^, using molecule ID 9790.

We simulated a sample of 10.0% H_2_O in phytantriol with lamellar symmetry (*L*_α_), created following the protocol described in ref. ^53^ in a simulation box with dimensions 5 × 5 × 3.25 nm. The system was equilibrated at 27 °C. Initial energy minimisation was performed using the steepest descent algorithm, followed by a 5 ps canonical (NVT) ensemble simulation during which the integration time step was gradually increased from 0.4 to 2.0 fs in increments of 0.4 fs.

Volume relaxation was then achieved via a 5 ns isothermal-isobaric (NPT) ensemble simulation using the C-rescale semi-isotropic barostat at a constant pressure of 1 bar. A final 1 ns NVT simulation was used to complete the equilibration. Temperature was maintained using a Langevin thermostat with a damping constant of 0.5 ps^−1^. Periodic boundary conditions were applied in all three dimensions. Lennard-Jones interactions and short-range electrostatics were treated with a cutoff at 1.2 nm, whereas the long-range electrostatics were implemented with particle mesh Ewald summation. The final integration time step was set to 2 fs, made possible by constraining covalent bonds involving hydrogen atoms using the LINCS algorithm. For simulations at lower temperatures (−3 to −83 °C, in 10 °C increments), initial configurations were taken from equilibrated higher-temperature systems. These systems were equilibrated using the same final two steps as above (5 ns NPT followed by 1 ns NVT). The stability of the system was improved with the implementation of a constraint with an elastic constant of 20 kJ mol^−1^ nm^−2^ in the lipid heads throughout all simulations. Each system was simulated in five independent runs.

Production simulations were performed in the NVT ensemble at 1 µs for all temperatures. Trajectory data were sampled every 300 ps. The MS*D*_∥_ is defined as the squared displacement of the position of the oxygen atoms of the water molecules parallel to the membrane mid-plane, averaged over all water molecules:

9 $$\,{{\rm{MS}}}{D}_{\parallel }=\left\langle {\left|{r}_{\parallel }\left(t\right)-{r}_{\parallel }\left(0\right)\right|}^{2}\right\rangle$$

where ${r}_{\parallel }(t)$ is the position of the oxygen atom of the water molecules at time $t$ and ${r}_{\parallel }(0)$ is the reference position of the oxygen atom of the water molecules at the beginning of the simulation. The average displacement at 1 µs is calculated from the average of the square root of MS*D*_∥_ at $t$ = 999.3 ± 0.3 ns.

### Reporting summary

Further information on research design is available in the Nature Portfolio Reporting Summary linked to this article.

## Supplementary information

Supplementary Information

Description of Additional Supplementary Information

Supplementary Data 1

Reporting Summary

## Source data

Source Data

Transparent Peer Review file

## Data Availability

All data that support the findings of this study are available within the article and the Supplementary Information. Source data are provided with this paper. All initial and final molecular dynamics configurations are provided in Supplementary_Data_1.zip. Source data are provided with this paper.

## References

[1] Ball, P. Water — an enduring mystery. *Nature* **452**, 291–292 (2008). 18354466 10.1038/452291a

[2] Pettersson, L. G. M., Henchman, R. H. & Nilsson, A. Water—the most anomalous liquid. *Chem. Rev.* **116**, 7459–7462 (2016). 27405667 10.1021/acs.chemrev.6b00363

[3] Rescigno, M. et al. Observation of plastic ice VII by quasi-elastic neutron scattering. *Nature* **640**, (2025). 10.1038/s41586-025-08750-4 PMC12003197 39938568

[4] Salzmann, C. G., Loveday, J. S., Rosu-Finsen, A. & Bull, C. L. Structure and nature of ice XIX. *Nat. Commun*. **12**, (2021). 10.1038/s41467-021-23399-z PMC8155070 34039987

[5] Rosu-Finsen, A. et al. Medium-density amorphous ice. *Science* **379**, 474–478 (2023). 36730416 10.1126/science.abq2105

[6] Mishima, O., Calvert, L. D. & Whalley, E. An apparently first-order transition between two amorphous phases of ice induced by pressure. *Nature* **314**, 76–78 (1985).

[7] Bogdan, A., Molina, M. J., Tenhu, H., Mayer, E. & Loerting, T. Formation of mixed-phase particles during the freezing of polar stratospheric ice clouds. *Nat. Chem.* **2**, 197–201 (2010). 21124476 10.1038/nchem.540

[8] Clarke, A. et al. A low temperature limit for life on Earth. *PLoS One* **8**, (2013). 10.1371/journal.pone.0066207 PMC3686811 23840425

[9] Sciortino, F., Gallo, P., Tartaglia, P. & Chen, S. H. Supercooled water and the kinetic glass transition. *Phys. Rev. E* **54**, 6331–6343 (1996). 10.1103/physreve.54.6331 9965854

[10] Handle, P. H., Loerting, T. & Sciortino, F. Supercooled and glassywater: Metastable liquid(s), amorphous solid(s), and a no-man’s land. *Proc. Natl. Acad. Sci. USA* **114**, 13336–13344 (2017). 29133419 10.1073/pnas.1700103114 PMC5754753

[11] Mezzenga, R. Challenges and opportunities from water under soft nanoconfinement. *Front. Soft Matter* **3**, 1–7 (2023).

[12] Velikov, V., Borick, S. & Angell, C. A. The glass transition of water, based on hyperquenching experiments. *Science* **294**, 2335–2338 (2001). 11743196 10.1126/science.1061757

[13] Kim, K. H. et al. Maxima in the thermodynamic response and correlation functions of deeply supercooled water. *Science* **358**, 1589–1593 (2017). 29269472 10.1126/science.aap8269

[14] Mishima, O. & Stanley, H. E. The relationship between liquid, supercooled and glassy water. *Nature* **396**, 329–335 (1998).

[15] Langham, E. & Mason, B. The heterogeneous and homogeneous nucleation of supercooled water. *Proc. R. Soc. Lond. A* **247**, 493–504 (1958).

[16] Smart, A. G. Supercooled water survives in no-man’s-land. *Phys. Today* **70**, 18–21 (2017).

[17] Debenedetti, P. G. & Stanley, H. E. Supercooled and glassy water. *Phys. Today* **56**, 40–46 (2003).

[18] Melillo, J. H. et al. Complexity of confined water vitrification and its glass transition temperature. *Proc. Natl. Acad. Sci. USA* **121**, 1–10 (2024). 10.1073/pnas.2407030121 PMC11474062 39356669

[19] Cerveny, S., Mallamace, F., Swenson, J., Vogel, M. & Xu, L. Confined water as model of supercooled water. *Chem. Rev.* **116**, 7608–7625 (2016). 26940794 10.1021/acs.chemrev.5b00609

[20] Gallo, P. et al. Water: a tale of two liquids. *Chem. Rev.* **116**, 7463–7500 (2016). 27380438 10.1021/acs.chemrev.5b00750 PMC5424717

[21] Ruska, E. The development of the electron microscope and of electron microscopy. *Rev. Mod. Phys*. **59**, (1987).

[22] Salvati Manni, L. et al. Soft biomimetic nanoconfinement promotes amorphous water over ice. *Nat. Nanotechnol.* **14**, 609–615 (2019). 30962546 10.1038/s41565-019-0415-0

[23] Knight, A. W., Kalugin, N. G., Coker, E. & Ilgen, A. G. Water properties under nano-scale confinement. *Sci. Rep.* **9**, 1–12 (2019). 31160663 10.1038/s41598-019-44651-z PMC6546746

[24] Takamuku, T., Yamagami, M., Wakita, H., Masuda, Y. & Yamaguchi, T. Thermal property, structure, and dynamics of supercooled water in porous silica by calorimetry, neutron scattering, and NMR relaxation. *J. Phys. Chem. B* **101**, 5730–5739 (1997).

[25] Israelachvili, J. N. *Intermolecular and surface forces*. Academic Press, (2011).

[26] Debenedetti, P. G. & Stillinger, F. H. Supercooled liquids and the glass transition. *Nature* **410**, 259–267 (2001). 11258381 10.1038/35065704

[27] Franks, F. *Water: a matrix of life*. Royal Society of Chemistry, (2000).

[28] Malkin, T. L., Murray, B. J., Brukhno, A. V., Anwar, J. & Salzmann, C. G. Structure of ice crystallized from supercooled water. *Proc. Natl. Acad. Sci. USA* **109**, 1041–1045 (2012). 22232652 10.1073/pnas.1113059109 PMC3268266

[29] Yao, Y. et al. Designing cryo-enzymatic reactions in subzero liquid water by lipidic mesophase nanoconfinement. *Nat. Nanotechnol.* **16**, 802–810 (2021). 33941918 10.1038/s41565-021-00893-5

[30] Wood, K. et al. Coupling of protein and hydration-water dynamics in biological membranes. *Proc. Natl. Acad. Sci. USA* **104**, 18049–18054 (2007). 17986611 10.1073/pnas.0706566104 PMC2084294

[31] Bée, M. Quasielastic neutron scattering. *Principles and Applications in Solid State Chemistry, Biology, and Materials Science*. Adam Hilger (1988).

[32] Manni, L. S., Wood, K., Klapproth, A. & Warr, G. G. Inelastic neutron scattering and spectroscopy methods to characterise dynamics in colloidal and soft matter systems. *Adv. Colloid Interface Sci.* **326**, 103135 (2024). 38520888 10.1016/j.cis.2024.103135

[33] Wuttke, J., Petry, W., Coddens, G. & Fujara, F. Fast dynamics of glass-forming glycerol. *Phys. Rev. E* **52**, (1995). 10.1103/physreve.52.4026 9963875

[34] Angell, C. A., Ngai, K. L., McKenna, G. B., McMillan, P. F. & Martin, S. W. Relaxation in glassforming liquids and amorphous solids. *J. Appl. Phys.* **88**, 3113–3157 (2000).

[35] Singwi, K. S. & Sjölander, A. Diffusive Motions in Water and Cold Neutron Scattering. *Phys. Rev.* **119**, 863–871 (1960).

[36] Yepuri, N. R. et al. Deuterated phytantriol – A versatile compound for probing material distribution in liquid crystalline lipid phases using neutron scattering. *J. Colloid Interface Sci.* **534**, 399–407 (2019). 30245337 10.1016/j.jcis.2018.09.022

[37] Schwaab, G., Sebastiani, F. & Havenith, M. Ion hydration and ion pairing as probed by THz spectroscopy. *Angew. Chem. - Int. Ed.* **58**, 3000–3013 (2019). 10.1002/anie.201805261 30022575

[38] Tanaka, H. Roles of liquid structural ordering in glass transition, crystallization, and water’s anomalies. *J. Non-Cryst. Solids X* **13**, 100076 (2022).

[39] Nitzan, A. *Chemical dynamics in condensed phases: relaxation, transfer and reactions in condensed molecular systems*. Oxford University Press (2006).

[40] Salsbury, N. J., Darke, A. & Chapman, D. Deuteron magnetic resonance studies of water associated with phospholipids. *Chem. Phys. Lipids* **8**, 142–151 (1972). 5063097 10.1016/0009-3084(72)90026-6

[41] Lee, D.-K., Kwon, B. S. & Ramamoorthy, A. Freezing point depression of water in phospholipid membranes: a solid-state NMR study. *Langmuir* **24**, 13598–13604 (2008). 18991419 10.1021/la8023698 PMC2649677

[42] Goldmünz, E., Aserin, A. & Garti, N. Temperature-sensitive properties of occluded hydration centers in direct hexagonal (HI) mesophases. *Colloids Surf. A Physicochem. Eng. Asp.* **631**, 127709 (2021).

[43] Ulrich, A. S. & Grage, S. L. *Solid state NMR of polymers*. Elsevier, (1998).

[44] Gainaru, C. et al. Anomalously large isotope effect in the glass transition of water. *Proc. Natl. Acad. Sci. USA* **111**, 17402–17407 (2014). 25422420 10.1073/pnas.1411620111 PMC4267340

[45] Gallo, P., Sciortino, F., Tartaglia, P. & Chen, S.-H. Slow dynamics of water molecules in supercooled states. *Phys. Rev. Lett.* **76**, 2730–2733 (1996). 10060774 10.1103/PhysRevLett.76.2730

[46] Tavagnacco, L. et al. Water slowing down drives the occurrence of the low temperature dynamical transition in microgels. *Chem. Sci.* **15**, 9249–9257 (2024). 38903230 10.1039/d4sc02650k PMC11186305

[47] Lupi, L. & Gallo, P. Glassy dynamics of water in TIP4P/Ice aqueous solutions of trehalose in comparison with the bulk phase. *J. Chem. Phys.* **159**, 154504 (2023). 37850697 10.1063/5.0168933

[48] Kim, K. H. et al. Experimental observation of the liquid-liquid transition in bulk supercooled water under pressure. *Science* **370**, 978–982 (2020). 33214280 10.1126/science.abb9385

[49] Stillinger, F. H. A topographic view of supercooled liquids and glass formation. *Science* **267**, 1935–1939 (1995). 17770102 10.1126/science.267.5206.1935

[50] Sciortino, F. Potential energy landscape description of supercooled liquids and glasses. *J. Stat. Mech*. P05015 (2005).

[51] Keddie, J. L., Jones, R. A. L. & Cory, R. A. Size-dependent depression of the glass transition temperature in polymer films. *Europhys. Lett.* **27**, 59–64 (1994).

[52] Bachler, J. et al. Glass polymorphism in glycerol-water mixtures: II. Experimental studies. *Phys. Chem. Chem. Phys.* **18**, 11058–11068 (2016). 27044677 10.1039/c5cp08069j PMC4840991

[53] Zunzunegui-Bru, E. et al. Universality in the structure and dynamics of water under lipidic mesophase soft nanoconfinement. *ACS Nano* **18**, 21376–21387 (2024). 39088237 10.1021/acsnano.4c05857

[54] Franks, F. *The physics and physical chemistry of water*. Plenum Press, (1972).

[55] Barauskas, J. & Landh, T. Phase behavior of the phytantriol/water system. *Langmuir* **19**, 9562–9565 (2003).

[56] Australian Synchrotron. ScatterBrain version 2.82 (2021); https://asuserwiki.atlassian.net/wiki/spaces/UO/pages/358875147/scatterBrain+Software+Downloads.

[57] Li, N. Y. D., Perutková, Š, Iglič, A. & Rappolt, M. My first electron density map: A beginner’s guide to small angle X-ray diffraction. *Electrotech. Rev.* **84**, 69–75 (2017).

[58] Salvati Manni, L. et al. Unusual phosphatidylcholine lipid phase behavior in the ionic liquid ethylammonium nitrate. *J. Colloid Interface Sci.* **643**, 276–281 (2023). 37068361 10.1016/j.jcis.2023.03.161

[59] De Souza, N. R., Klapproth, A. & Iles, G. N. Emu: high-resolution backscattering spectrometer at ANSTO. *Neutron News* (2016).

[60] Yu, D., Mole, R., Noakes, T., Kennedy, S. & Robinson, R. Pelican — a time of flight cold neutron polarization analysis spectrometer at OPAL. *J. Phys. Soc. Jpn.* **82**, SA027 (2013).

[61] Arnold, O. et al. Mantid—Data analysis and visualization package for neutron scattering and μ SR experiments. *Nucl. Instrum. Methods Phys. Res. A Accel. Spectrometers Detect. Assoc. Equip.* **764**, 156–166 (2014).

[62] Swinehart, D. F. The Beer-Lambert Law. *J. Chem. Educ.* **39**, 333 (1962).

[63] Sharma, V., Böhm, F., Schwaab, G. & Havenith, M. The low frequency motions of solvated Mn(ii) and Ni(ii) ions and their halide complexes. *Phys. Chem. Chem. Phys.* **16**, 25101–25110 (2014). 25332014 10.1039/c4cp03989k

[64] Funke, S., Sebastiani, F., Schwaab, G. & Havenith, M. Spectroscopic fingerprints in the low frequency spectrum of ice (Ih), clathrate hydrates, supercooled water, and hydrophobic hydration reveal similarities in the hydrogen bond network motifs. *J. Chem. Phys.* **150**, 224505 (2019). 31202220 10.1063/1.5097218

[65] Fukasawa, T. et al. Relation between dielectric and low-frequency Raman spectra of hydrogen-bond liquids. *Phys. Rev. Lett.* **95**, 197802 (2005). 16384025 10.1103/PhysRevLett.95.197802

[66] van Meerten, S. G. J., Franssen, W. M. J. & Kentgens, A. P. M. ssNake: a cross-platform open-source NMR data processing and fitting application. *J. Magn. Reson.* **301**, 56–66 (2019). 30851666 10.1016/j.jmr.2019.02.006

[67] Lindahl, E., Hess, B. & van der Spoel, D. GROMACS 3.0: a package for molecular simulation and trajectory analysis. *Mol. Model. Annu.* **7**, 306–317 (2001).

[68] Abascal, J. L. F., Sanz, E., García Fernández, R. & Vega, C. A potential model for the study of ices and amorphous water: TIP4P/ice. *J. Chem. Phys.* **122**, 234511 (2005). 16008466 10.1063/1.1931662

[69] Malde, A. K. et al. An automated force field topology builder (ATB) and repository: version 1.0. *J. Chem. Theory Comput.* **7**, 4026–4037 (2011). 26598349 10.1021/ct200196m

