# Supplementary material for: Resolving liquid-to-glass transitions of water under soft nanoconfinement
Source: Nat Commun. 2026 May 8;17:6224. doi: 10.1038/s41467-026-72955-y (PMC13369869; doi:10.1038/s41467-026-72955-y)
Supplement: Supplementary file 1 — Supplementary Information [file 41467_2026_72955_MOESM1_ESM.pdf]

Supplementary Information

Resolving liquid-to-glass transitions of water under soft nanoconfinement

Patrick Züblin[1,†], Eva Zunzunegui-Bru[1,†], Livia Salvati Manni[2,3], Alice Klapproth[4], Richard Mole[4], Nageshwar Rao Yepuri[4], Syrine Khaled[5], Guillaume Pierre Laurent[5], Thierry Azaïs[5], Serena Rosa Alfarano[1], Jean-Blaise Brubach[6], Salvatore Assenza[7,8,9], Francesco Sciortino[10], Raffaele Mezzenga[1,11]*

[1] Department of Health Sciences and Technology, ETH Zürich, Zürich, Switzerland

[2] School of Chemistry and University of Sydney Nano Institute, The University of Sydney, Sydney, Australia

[3] Australian Nuclear Science and Technology Organisation, Clayton, Australia

[4] Australian Nuclear Science and Technology Organisation, Lucas Heights, Australia

[5] Sorbonne Université, CNRS, Laboratoire de Chimie de la Matière Condensée de Paris (LCMCP), Paris, France

[6] Synchrotron SOLEIL, CNRS, Saint-Aubin, France

[7] Departamento de Física Teórica de la Materia Condensada, Universidad Autónoma de Madrid, Madrid, Spain

[8] Condensed Matter Physics Center (IFIMAC), Universidad Autónoma de Madrid, Madrid, Spain

[9] Instituto Nicolás Cabrera, Universidad Autónoma de Madrid, Madrid, Spain

[10] Dipartimento di Fisica, Sapienza Università di Roma, Rome, Italy

[11] Department of Materials, ETH Zürich, Zürich, Switzerland

[†] These authors contributed equally: Patrick Züblin, Eva Zunzunegui-Bru

* raffaele.mezzenga@hest.ethz.ch

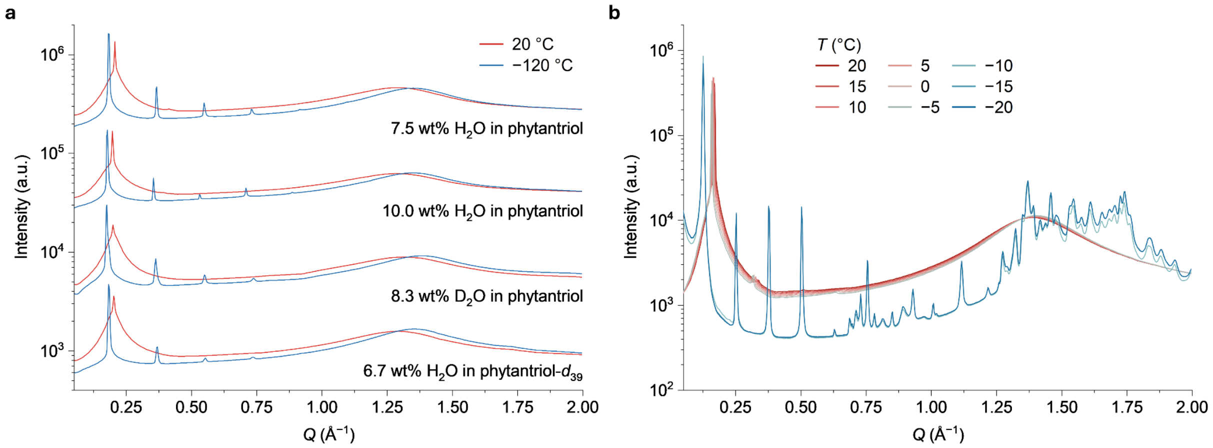

Supplementary Fig. 1. Low-temperature X-ray scattering data. a, SAXS/WAXS profiles of four mesophases of different compositions at 20 and −120 °C. The temperature rate was 1 °C min^{-1} and SAXS/WAXS frames were acquired within 1 s. Curves are offset vertically for clarity. **b**, SAXS/WAXS profiles of 7.5 wt% H_2O in Monoolein during cooling from 25 to −20 °C at 1 °C min^{-1}. The rapid transition from a mixed $L_2 + L_\alpha$ phase to a lamellar crystalline phase occurs between −5 and −10 °C. a.u., arbitrary units.

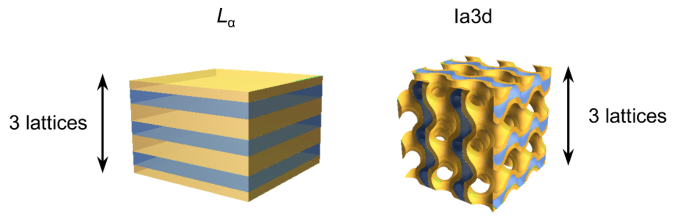

Supplementary Fig. 2. Mesophase symmetries. Schematics of the lamellar (L_α) and gyroid cubic (Ia3d) symmetries. The cubic phase contains two non-communicating but interpenetrating water channel networks, only one of which is shown here. The spatial extent of three lattice units is indicated. The schematics of were created using Wolfram Mathematica version 11.0.

S3. Supplementary Note on the freezing of water in 15.0 wt% H_2O in phytantriol

The freezing of water in the mesophase of 15.0 wt% H_2O in phytantriol between −40 and −50 °C was accompanied by two simultaneous lamellar phase SAXS/WAXS patterns (Supplementary Fig. 3). This observation, together with the abrupt drop in the repeat spacing of lamellae (lattice size) (ref. 1), indicates that water partially migrates out of the intermembrane regions, where it subsequently crystallises into ice external to the organised membrane stacks. Residual, non-crystalline water remains associated with the hydration the lipidic bilayers.

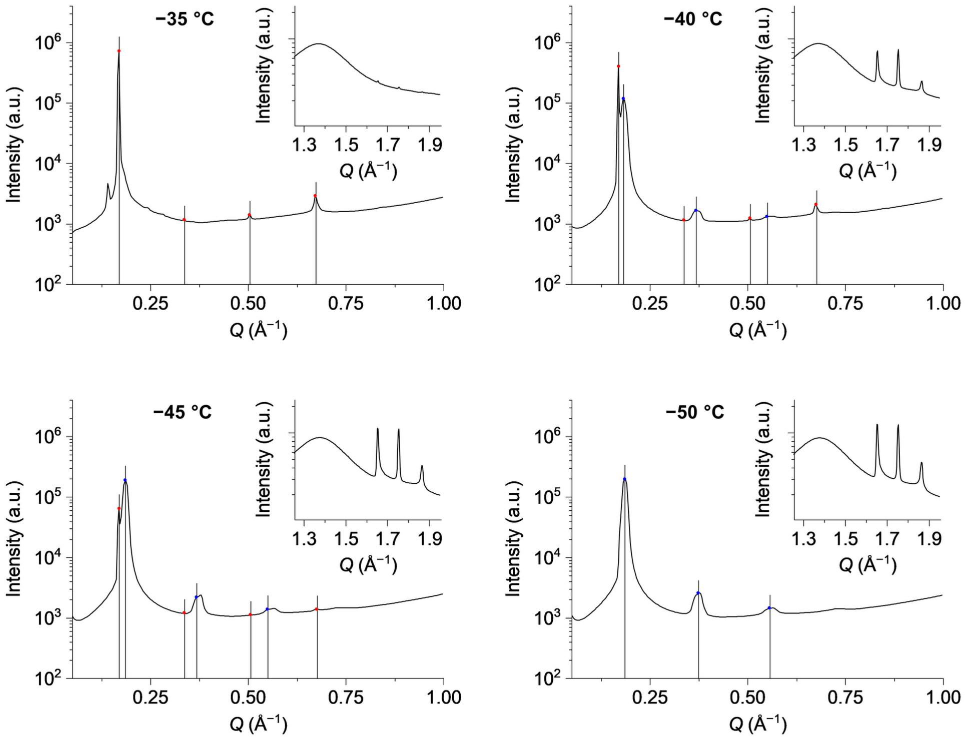

Supplementary Fig. 3. SAXS/WAXS profiles of 15.0 wt% H_2O in phytantriol during cooling. The Bragg peaks of the two lamellar phases are marked in red and blue. The insets show a zoom of hexagonal ice Bragg reflections. The data was collected at the SAXS/WAXS beamline of the Australian Synchrotron. The cooling rate was 1 °C min^{-1} and SAXS/WAXS frames were acquired within 1 s. a.u., arbitrary units.

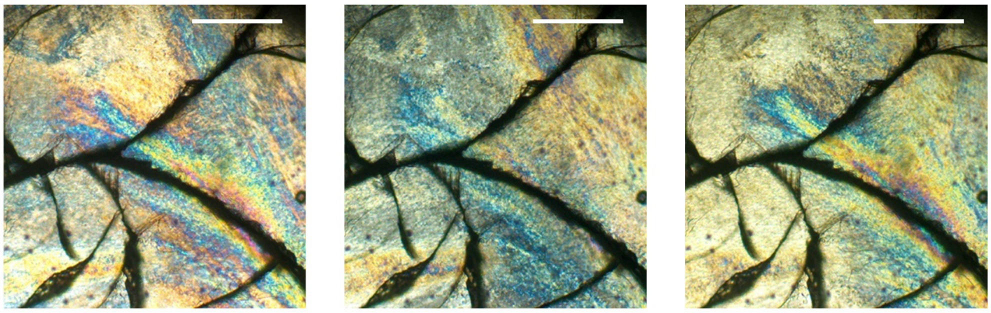

Supplementary Fig. 4. Mesophase of 7.5 wt% H_2O in phytantriol at −130 °C under crossed polarizers. Owing to the birefringent properties of the lamellar phase structure, we can visually confirm the physical nature of the cracks by rotating the polarizers. Scale bars, 100 µm.

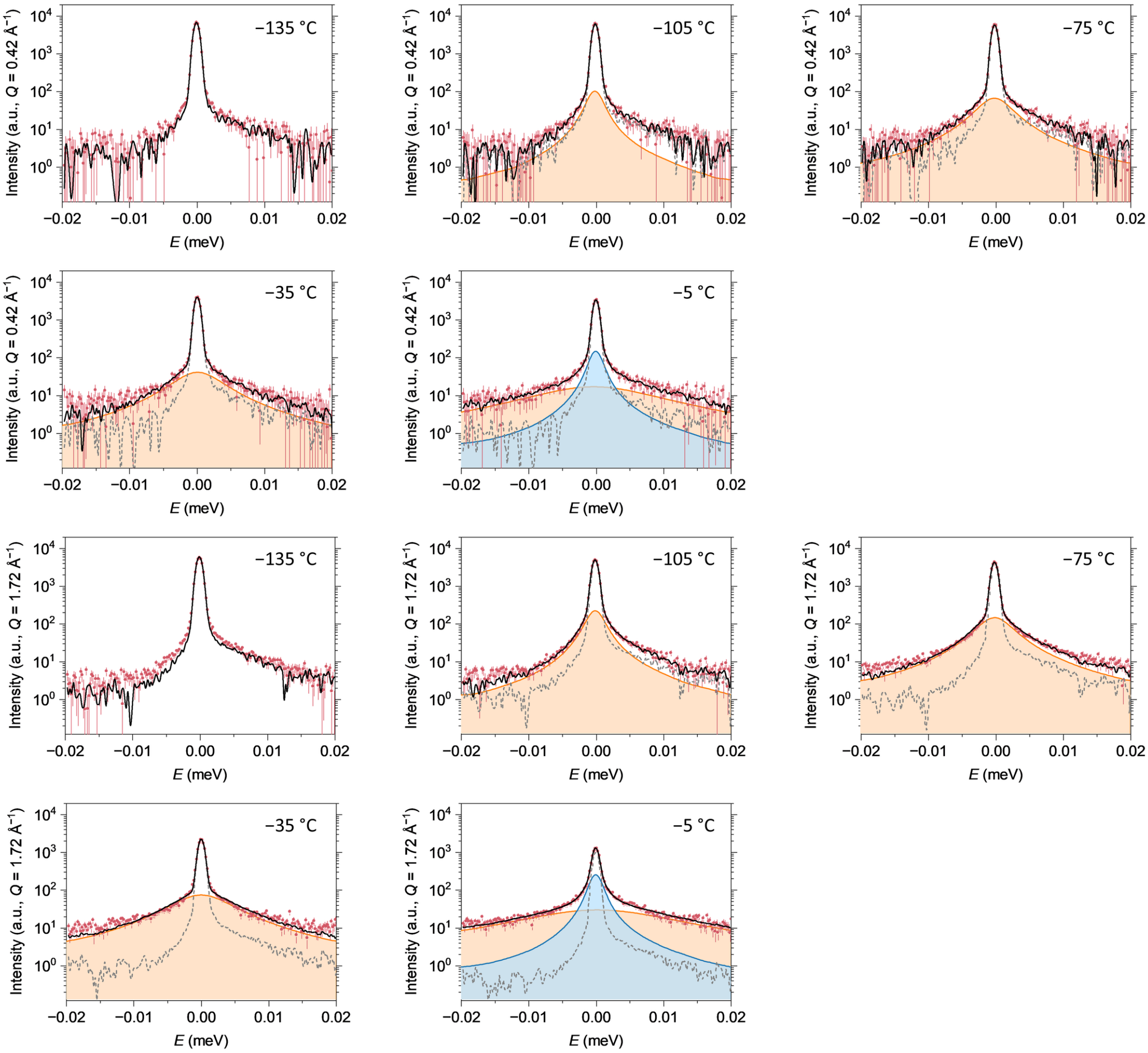

Supplementary Fig. 5. Fitting of the QENS data of 7.5 wt% H_2O in phytantriol at fixed *Q*-values of 0.42 and 1.72 Å^{-1} from −135 to −5 °C. Experimental data is shown in red, the instrument resolution as a grey dashed line and the total fit as a black solid line. The Lorentzian contributions are shown in orange (phytantriol) and blue (water). Data was collected on the Emu backscattering spectrometer at the Australian Centre for Neutron Scattering. Error bars represent propagated counting statistics (square root of neutron counts) as mean ± standard deviation and do not reflect variability from independent measurements (n = 1).

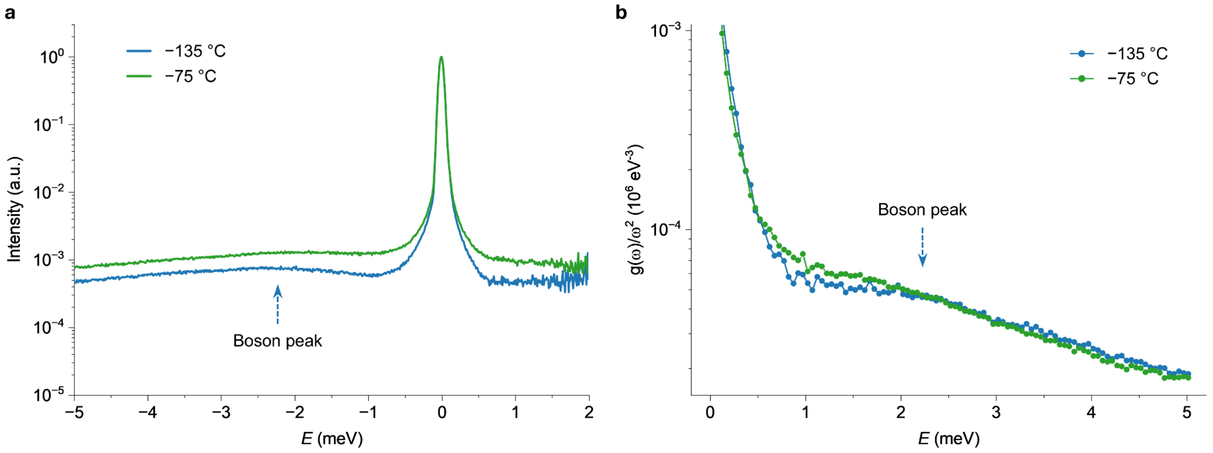

Supplementary Fig. 6. Confirmation of a boson peak. a, *Q*-integrated QENS spectra of 7.5 wt% H_2O in phytantriol at −135 and −75 °C on a ~1 to ~20 ps time scale. **b**, Reduced vibrational density of states of the intensity data from **a**, plotted as $g(\omega)/\omega^2$. The excess in intensity appears at 2.2 meV and −135 °C but is obscured at −75 °C by the onset of QENS broadening. Data was collected on the Pelican time-of-flight spectrometer at the Australian Centre for Neutron Scattering. a.u., arbitrary units.

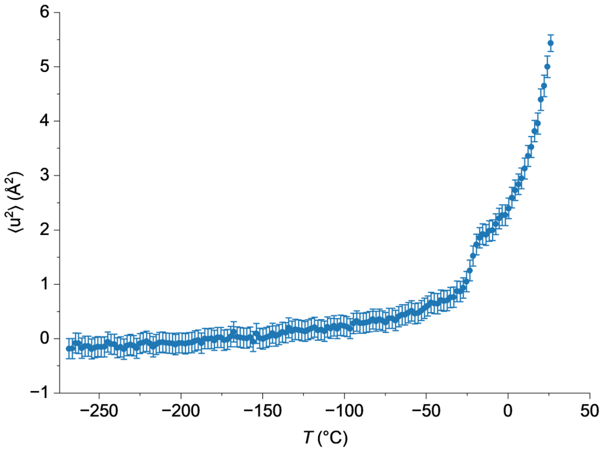

Supplementary Fig. 7. Apparent mean-squared displacement $\langle u^2 \rangle$ vs. temperature of 6.7 wt% H_2O in phytantriol-d_{39} derived from the elastic FWS data shown in Fig. 3g. The T_g of water is evident at around −25 °C by a sudden change in the slope of the mean-squared displacement. Data were recorded during a temperature ramp from −269 to 27 °C at a rate of 0.5 °C min^{-1}, ensuring near-equilibrium conditions and minimising thermal lag. The *Q*-range considered for the analysis was 0.32–0.92 $Å^{-1}$. The error bars denote the mean ± standard deviation of the neutron counts and do not reflect variability from independent measurements (n = 1).

Supplementary Table 1. Coherent and incoherent neutron scattering cross-sections (in percent) for the three samples from QENS experiments, categorised by molecular origin.

Sample	% of total σ_{coh}				% of total σ_{incoh}			
	H_2O	D_2O	Phyt-head	Phyt-tail	H_2O	D_2O	Phyt-head	Phyt-tail
7.5 wt% H_2O in phytantriol	5.5	0	24.3	70.2	6.6	0	20.0	73.4
8.3 wt% D_2O in phytantriol	0	10.4	23.0	66.6	0	0.1	21.4	78.5
6.7 wt% H_2O in phytantriol-d_{39}	3.2	0	20.6	76.2	42.4	0	45.4	12.2

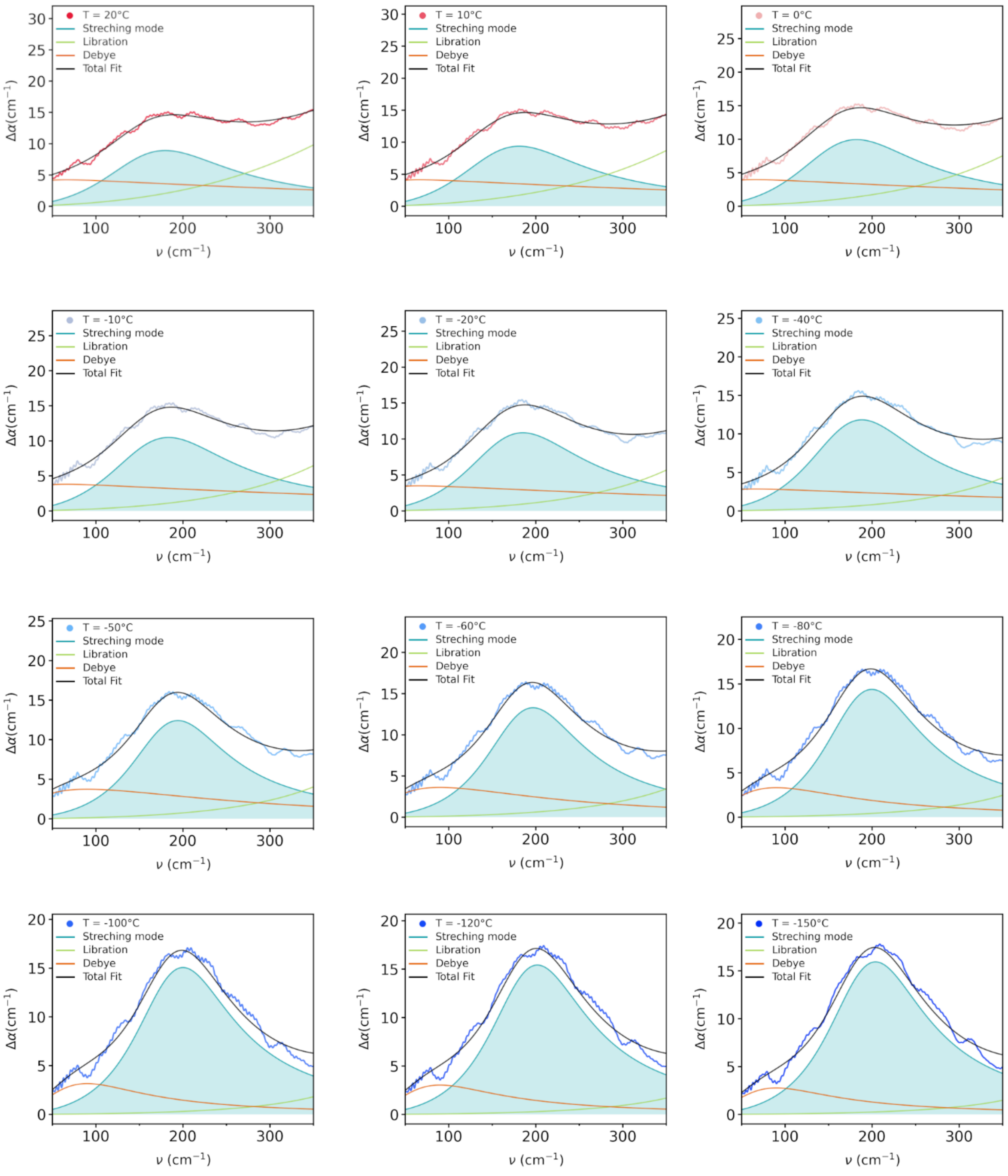

Supplementary Fig. 8. THz absorption spectroscopy. Modified damped harmonic oscillator fits of 10.0 wt% H_2O in phytantriol across all investigated temperatures. The intermolecular stretching mode is highlighted with a filled turquoise line, while the Debye and librational modes are shown in orange and green, respectively. The total fit is represented by a black line. Experimental data were collected at the AILES beamline of the SOLEIL synchrotron and are shown as coloured circles, ranging from red to blue with decreasing temperature.

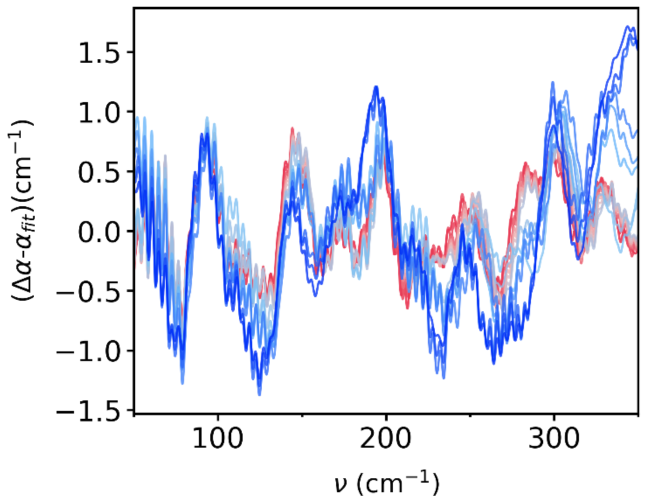

Supplementary Fig. 9. THz Absorption Spectroscopy. Residual errors from the spectral fit of the full THz signal, as presented in the Methods section in Eq. (6), at all investigated temperatures for 10.0 wt% H_2O in phytantriol. These residuals reflect the etalon effects present in the experimental absorption spectra.

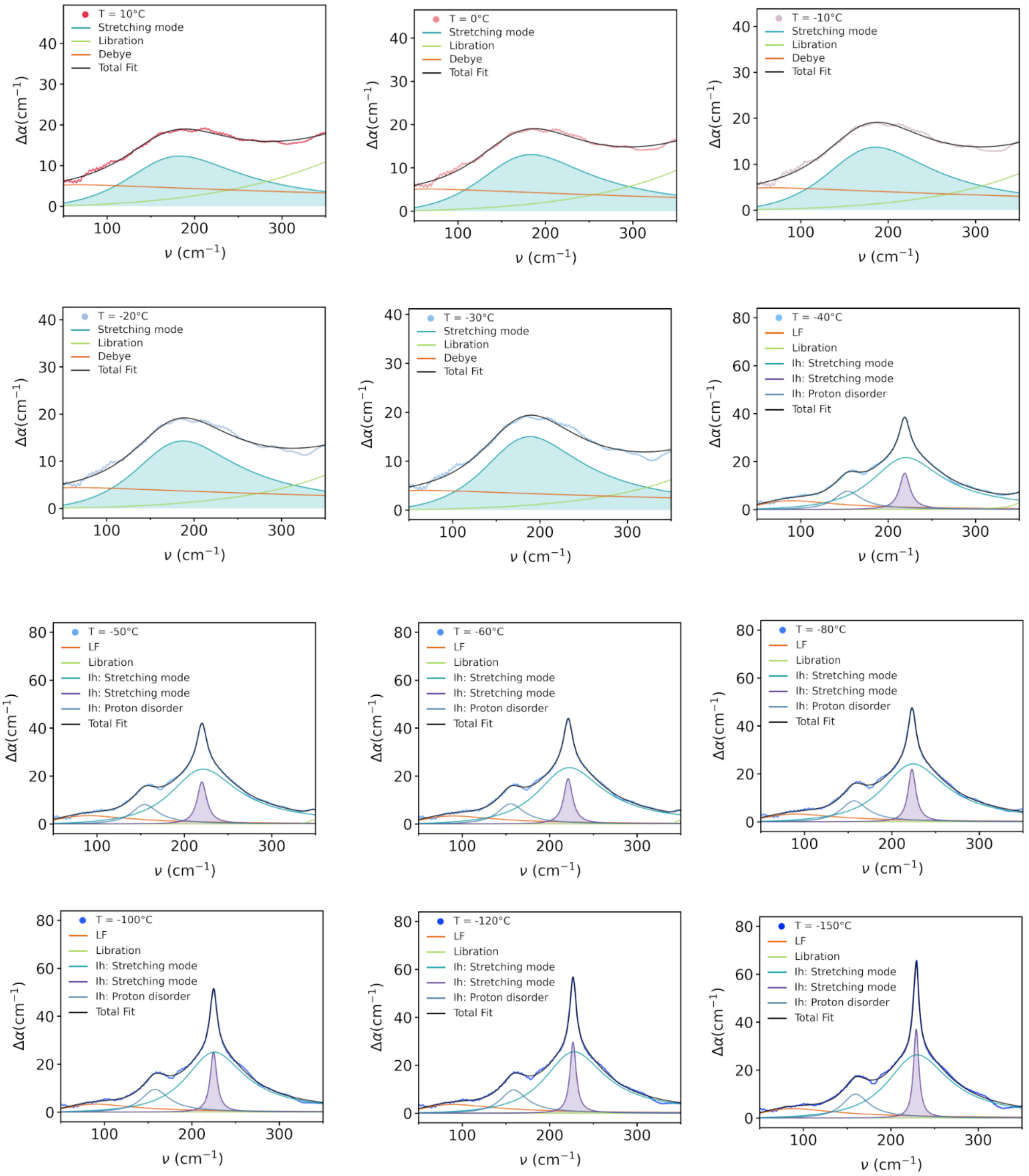

Supplementary Fig. 10. THz absorption spectroscopy. Modified damped harmonic oscillator fits of 15.0 wt% H_2O in phytantriol across all investigated temperatures. For measurements above −40 °C, the intermolecular stretching mode is highlighted with a filled turquoise line, while the Debye and librational modes are shown in orange and green, respectively. For measurements at or below −40 °C, corresponding to the formation of hexagonal ice, two intermolecular modes and the proton disordered water are included in the fit, shown in turquoise, filled purple and dark blue, respectively. The Debye mode remains in orange. The total fit is represented by a black line. Experimental data were collected at the AILES beamline of the SOLEIL synchrotron and are shown as coloured circles, ranging from red to blue with decreasing temperature.

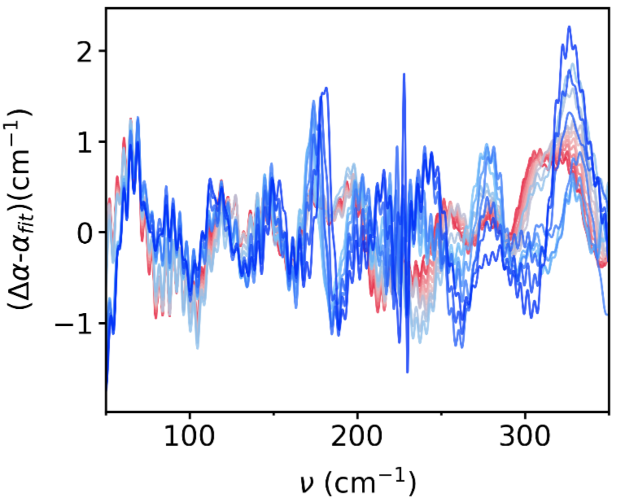

Supplementary Fig. 11. THz Absorption Spectroscopy. Residual errors from the spectral fit of the full THz signal, as presented in the Methods section in Eq. (6), at all investigated temperatures for 15.0 wt% H_2O in phytantriol. These residuals reflect the etalon effects present in the experimental absorption spectra.

Supplementary Table 2. Fitting parameters (given in cm^{-1}) of the THz spectra for 10.0 wt% H_2O in phytantriol, as shown in Fig. 4a,b in the main text. The vibrational modes of water are referred to as 'stret' (stretching), 'lib' (libration) and 'LF' (Debye low-frequency mode).

T (°C)	$\nu_{0,stret}$	$a_{0,stret}$	$\omega_{0,stret}$	$\nu_{0,lib}$	$a_{0,lib}$	$\omega_{0,lib}$	$\nu_{0,LF}$	$a_{0,LF}$	$\omega_{0,LF}$
20	176.74 ± 0.25	145.20 ± 0.66	585.88 ± 3.54	650.20 ± 3.84	375.13 ± 2.91	2088.78 ± 26.94	66.16 ± 0.38	44.78 ± 0.37	234.84 ± 7.95
10	178.62 ± 0.23	152.14 ± 0.58	584.04 ± 3.21	619.78 ± 2.45	348.00 ± 1.82	1753.26 ± 18.61	66.61 ± 0.37	43.83 ± 0.36	229.20 ± 7.59
0	180.23 ± 0.21	159.04 ± 0.47	577.81 ± 2.59	589.94 ± 1.29	318.85 ± 1.03	1454.47 ± 9.91	67.38 ± 0.36	42.49 ± 0.32	225.80 ± 6.89
−10	182.10 ± 0.19	164.98 ± 0.44	570.81 ± 2.27	569.68 ± 0.89	296.70 ± 0.67	1233.26 ± 7.05	68.19 ± 0.36	41.21 ± 0.30	231.61 ± 6.97
−20	184.86 ± 0.21	166.02 ± 0.56	546.41 ± 2.08	560.77 ± 0.67	282.40 ± 0.58	1106.40 ± 5.50	69.25 ± 0.48	38.29 ± 0.29	271.44 ± 10.19
−40	190.29 ± 0.23	167.67 ± 1.49	506.17 ± 2.60	546.30 ± 0.50	254.20 ± 0.41	888.59 ± 3.76	78.58 ± 1.55	33.27 ± 0.47	418.81 ± 32.06
−50	194.36 ± 0.11	155.94 ± 0.87	440.00 ± 2.46	545.29 ± 0.43	240.86 ± 0.63	808.40 ± 4.63	90.00 ± 2.03	46.83 ± 0.43	880.27 ± 28.94
−60	196.93 ± 0.10	166.83 ± 1.21	440.00 ± 2.72	545.00 ± 0.39	232.15 ± 0.51	748.56 ± 4.19	90.00 ± 1.99	45.31 ± 0.49	726.93 ± 31.08
-80	199.76 ± 0.10	180.62 ± 1.75	440.00 ± 3.08	545.00 ± 0.38	213.98 ± 0.44	642.14 ± 3.34	90.00 ± 2.19	41.65 ± 0.56	573.24 ± 37.23
-100	200.16 ± 0.17	189.21 ± 1.54	440.00 ± 2.49	545.00 ± 0.43	194.24 ± 0.41	569.10 ± 2.95	90.00 ± 1.71	39.64 ± 0.48	463.80 ± 29.23
−120	201.94 ± 0.16	193.76 ± 1.61	440.00 ± 2.59	545.00 ± 0.43	194.28 ± 0.48	547.31 ± 3.04	90.00 ± 1.89	38.00 ± 0.53	478.73 ± 33.10
−150	204.05 ± 0.16	200.29 ± 1.53	440.00 ± 2.46	545.00 ± 0.42	195.21 ± 0.47	505.86 ± 2.81	90.00 ± 1.95	34.68 ± 0.46	469.54 ± 34.75

Supplementary Table 3. Fitting parameters (given in cm^{-1}) of the THz spectra for 15.0 wt% H_2O in phytantriol, at temperatures where liquid water is present and as shown in Supplementary Fig. 12. The vibrational modes of water are referred to as 'stret' (stretching), 'lib' (libration) and 'LF' (Debye low-frequency mode).

T (°C)	$\nu_{0,stret}$	$a_{0,stret}$	$\omega_{0,stret}$	$\nu_{0,lib}$	$a_{0,lib}$	$\omega_{0,lib}$	$\nu_{0,LF}$	$a_{0,LF}$	$\omega_{0,LF}$
10	188.27 ± 0.28	162.65 ± 4.22	506.66 ± 6.15	594.72 ± 1.34	432.74 ± 1.29	1587.09 ± 11.18	88.07 ± 2.83	72.01 ± 1.67	541.03 ± 44.02
0	188.73 ± 0.25	173.21 ± 3.98	501.63 ± 5.53	567.74 ± 0.69	391.11 ± 0.74	1324.67 ± 6.42	88.22 ± 2.76	69.04 ± 1.57	535.96 ± 42.87
−10	190.23 ± 0.23	182.04 ± 3.49	491.00 ± 4.75	554.49 ± 0.53	361.33 ± 0.57	1157.46 ± 4.96	88.96 ± 2.51	65.73 ± 1.35	525.03 ± 38.93
−20	191.77 ± 0.22	190.33 ± 3.13	474.74 ± 4.21	546.79 ± 0.52	340.57 ± 0.53	1038.46 ± 4.57	89.84 ± 2.43	59.96 ± 1.17	510.76 ± 37.81
−30	192.78 ± 0.22	200.89 ± 2.32	458.97 ± 3.14	545.00 ± 0.47	322.69 ± 0.44	973.90 ± 4.11	90.00 ± 1.85	55.33 ± 0.86	465.16 ± 28.74

Supplementary Table 4. Fitting parameters (given in cm^{-1}) of the THz spectra for 15.0 wt% H_2O in phytantriol, at temperatures where hexagonal ice is present and as shown in Supplementary Fig. 12. The vibrational modes of hexagonal ice are referred to as 'stret1' (stretching mode 1), 'stret2' (stretching mode 2) and 'prot' (proton disordered water molecules).

T (°C)	$\nu_{0,stret1}$	$a_{0,stret1}$	$\omega_{0,stret1}$	$\nu_{0,stret2}$	$a_{0,stret2}$	$\omega_{0,stret2}$	$\nu_{0,prot}$	$a_{0,prot}$	$\omega_{0,prot}$
−40	220.40 ± 0.08	272.00 ± 1.07	310.89 ± 1.98	219.02 ± 0.03	190.13 ± 1.13	46.22 ± 0.46	153.74 ± 0.11	95.35 ± 0.87	119.92 ± 1.74
−50	221.50 ± 0.07	287.41 ± 0.92	294.78 ± 1.08	220.11 ± 0.02	220.30 ± 0.95	42.26 ± 0.35	154.49 ± 0.10	101.42 ± 0.72	115.62 ± 1.46
−60	222.54 ± 0.07	295.34 ± 0.89	286.70 ± 1.00	221.17 ± 0.02	237.62 ± 0.96	38.91 ± 0.31	155.32 ± 0.10	105.00 ± 0.72	116.38 ± 1.44
−80	224.38 ± 0.08	303.38 ± 1.06	279.60 ± 2.49	223.03 ± 0.02	275.40 ± 1.32	34.39 ± 0.30	156.63 ± 0.11	110.03 ± 0.91	115.77 ± 1.67
−100	225.83 ± 0.08	313.89 ± 1.14	259.58 ± 2.30	224.80 ± 0.02	313.89 ± 1.39	29.47 ± 0.25	157.84 ± 0.11	119.08 ± 0.89	120.78 ± 1.68
−120	227.94 ± 0.08	321.70 ± 1.20	264.07 ± 2.34	226.77 ± 0.01	372.32 ± 1.49	27.04 ± 0.21	158.68 ± 0.11	121.58 ± 0.99	117.15 ± 1.74
−150	230.27 ± 0.10	330.97 ± 1.27	268.94 ± 2.48	229.06 ± 0.01	467.11 ± 1.77	23.49 ± 0.17	159.66 ± 0.13	125.22 ± 1.15	115.78 ± 1.96

Supplementary Table 5. Fitting parameters (given in cm^{-1}) of the THz spectra for 15.0 wt% H_2O in phytantriol, at temperatures where hexagonal ice is present and as shown in Supplementary Fig. 12. The vibrational modes of water are referred to as 'LF' (Debye low-frequency mode) and 'lib' (libration).

T (°C)	$\nu_{0,LF}$	$a_{0,LF}$	$\omega_{0,LF}$	$\nu_{0,lib}$	$a_{0,lib}$	$\omega_{0,lib}$
−40	90.00 ± 0.70	45.86 ± 0.28	346.20 ± 11.35	373.96 ± 12.79	28.04 ± 15.21	115.58 ± 14.60
−50	90.00 ± 0.68	43.41 ± 0.28	336.16 ± 11.04	347.36 ± 0.34	21.18 ± 0.79	133.62 ± 12.96
−60	90.00 ± 0.69	41.80 ± 0.29	328.96 ± 11.09	347.67 ± 0.27	18.14 ± 0.99	122.25 ± 22.59
−80	90.00 ± 0.77	40.38 ± 0.39	326.38 ± 12.68	679.24 ± 61.27	67.17 ± 52.25	100.16 ± 45.32
−100	90.00 ± 0.71	41.63 ± 0.40	299.85 ± 11.03	763.32 ± 26.97	10.42 ± 10.44	130.07 ± 61.35
−120	90.00 ± 0.77	45.70 ± 0.41	324.25 ± 12.63	690.61 ± 14.89	89.55 ± 12.49	132.07 ± 42.85
−150	88.22 ± 0.83	49.09 ± 0.46	336.48 ± 14.73	583.38 ± 50.03	48.77 ± 78.91	230.06 ± 15.05

Supplementary Table 6. Parameters of the sigmoidal fit of the intermolecular stretching mode frequency of H_2O presented in Fig. 4b in the main text.

$a(\mathrm{cm^{-1}K^{-1}})$	$b(\mathrm{cm^{-1}})$	$c(\mathrm{cm^{-1}})$	$x_0(\mathrm{K})$	$c_0(\mathrm{K})$
-0.052	-18.433	210.27	241.58	16.36

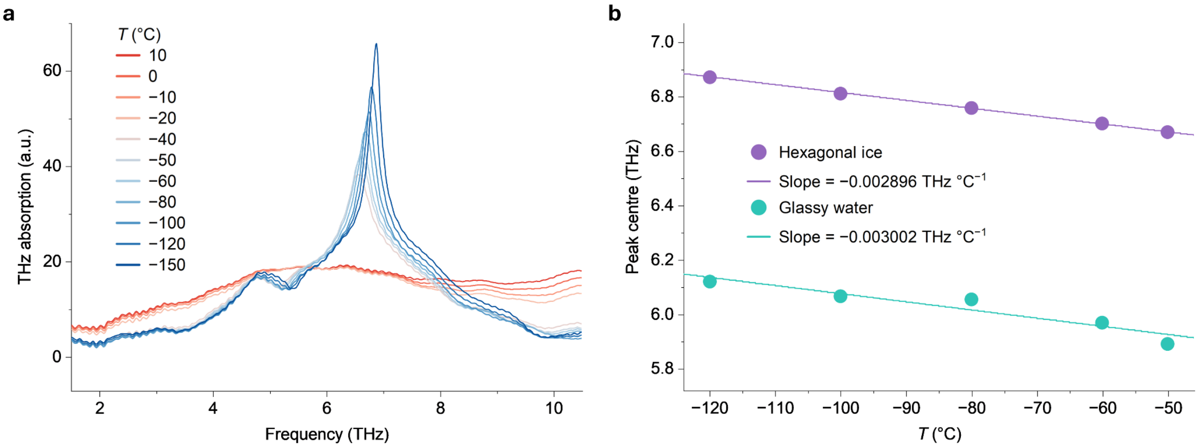

Supplementary Fig. 12. THz absorption spectroscopy of 15.0 wt% H_2O in phytantriol. a, Selection of isolated water THz absorption spectra upon cooling from 10 to −150 °C. The nanoconfined water partially freezes between −20 and −40 °C, agreeing with SAXS/WAXS measurements (Fig. 2b, inset). The crystalline ice peak shows a blueshift upon further cooling. The temperature rate was 1 °C min^{-1}. **b**, Centre frequency of the intermolecular stretching mode in glassy water and hexagonal ice as a function of temperature, shown as turquoise and purple circles, respectively. Both data sets are fitted with linear functions (solid lines) that exhibit the same slope. This indicates a comparable blueshift behaviour, i.e. an increase in the rigidity of the network, in these kinetically arrested systems. a.u., arbitrary units.

S13. Supplementary Note on ^{2}H solid-state NMR spectroscopy

The ^{2}H NMR spectra were obtained with an equilibration time of 20 min prior to the NMR acquisition at each specified temperature (Supplementary Fig. 13a). Deuterium possesses a nuclear spin of $I = 1$ and, in static condition, the ^{2}H NMR resonance exhibits typical line shapes dominated by the quadrupolar interaction. Simulation of ^{2}H NMR spectra allows the determination of the quadrupolar constant C_Q that is directly related to the dynamics of the spin system. For a sample of 8.3 wt% D_2O in phytantriol, four distinct motional regimes are evidenced. (*i*) At 20 °C, D_2O molecules are in a free bulk state, in which fast isotropic reorientation averages out the quadrupolar coupling, leading to a single isotropic resonance. (*ii*) From 10 °C to −20 °C, D_2O molecules remain in a liquid-like state but experience a so-called fast anisotropic motional regime leading to a typical Pake doublet of small C_Q (Supplementary Fig. 13b), consistent with previous studies on water in lipidic mesophases[2,3]. In this regime, the C_Q value increases from 6.5 to 9.5 kHz, evidencing a decrease in the motional averaging of the quadrupolar coupling as the temperature is lowered from 10 to −20 °C. (*iii*) The NMR signal is lost from −30 to −50 °C because the dynamics of D_2O molecules slow down to an intermediate motional regime, where the correlation rate τ_C is equivalent to the quadrupole splitting ($\tau_C \approx 1/\Delta\nu_Q$) and can be estimated as $\tau_C \approx 5 \times 10^{-6}$ s for a quadrupole splitting of ~200 kHz. In this situation, the transverse relaxation time T_2 is fast enough to prevent ^{2}H NMR signal acquisition. Here, the temperature range of such an intermediate regime is surprisingly long[4,5]. (*iv*) Finally, at −60 °C and below, a broad signal ($C_Q \approx 198$ kHz) is recovered which is similar to the ^{2}H NMR spectrum of D_2O ice at −80 °C (Supplementary Fig. 13c), indicating that the D_2O molecules confined in phytantriol are in a slow motional regime ($\tau_C \gg 5 \times 10^{-6}$ s) and effectively rigid on the NMR time scale. Thus, the transition from a liquid-like state to rigid-like state occurs between −60 °C < T_g < −20 °C.

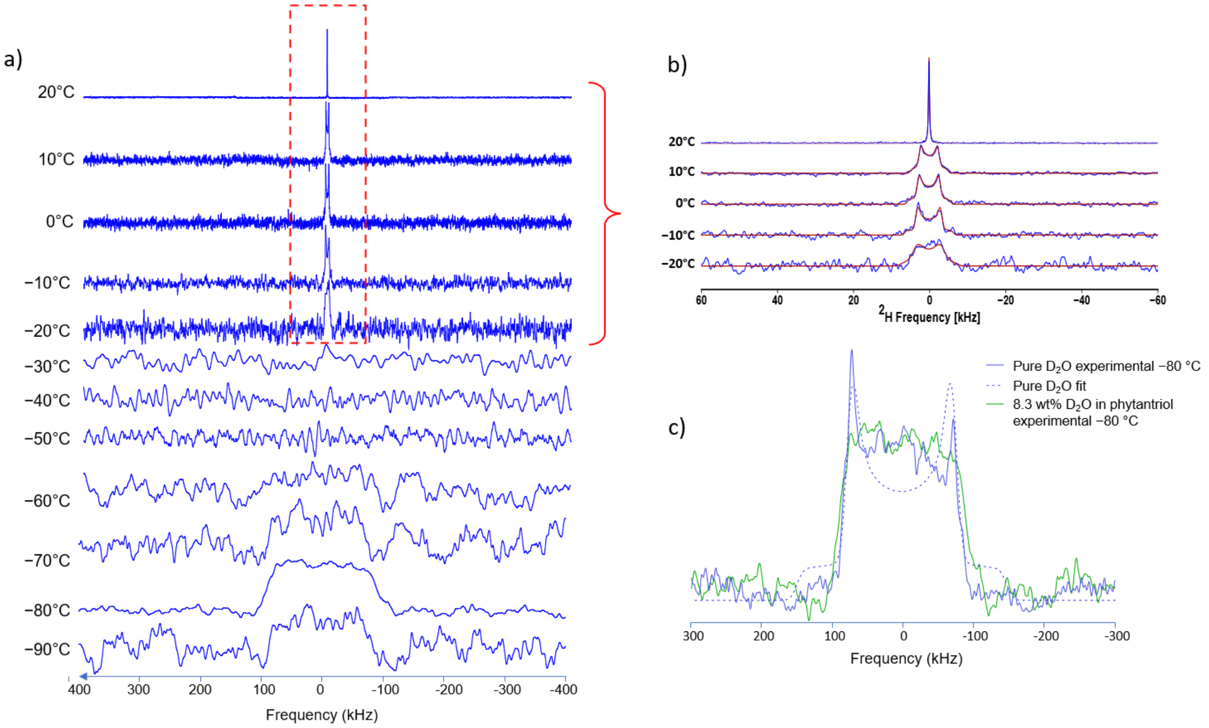

Supplementary Fig. 13. ^{2}H solid-state NMR spectroscopy. a) ^{2}H ssNMR spectra of 8.3 wt% D_2O in phytantriol between 20 and −90 °C. b) ^{2}H ssNMR spectra in the fast motional regime with the corresponding fitting in red with the following parameters: $\eta_Q = 0$ and C_Q = 6.5, 7.5, 8.6, 9.5 kHz at 10, 0, −10, −20 °C, respectively. c) Comparison of the ^{2}H ssNMR spectra of 8.3 wt% D_2O in phytantriol and pure D_2O at −80 °C showing the similar quadrupolar line shape where η_Q = 0 and C_Q = 198 kHz.

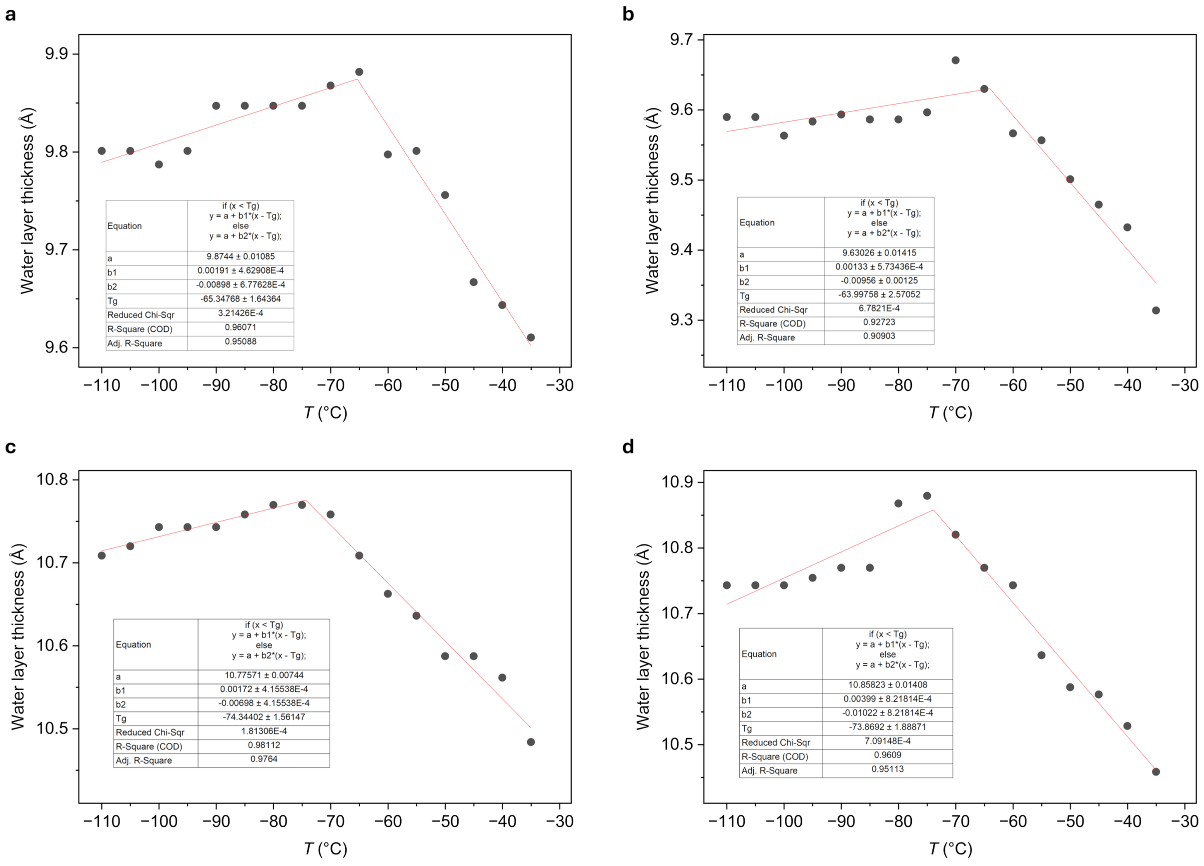

Supplementary Fig. 14. Piecewise linear fit to SAXS/WAXS data. Fit details of the piecewise linear regression applied to the data presented in Figure 4g. The data show the water layer thickness as a function of temperature in a sample of 8.3 wt% D_2O in phytantriol during the cooling (a) and heating cycle (b) and in a sample of 6.7 wt% H_2O in phytantriol-d_{39} during the cooling (c) and heating cycle (d).

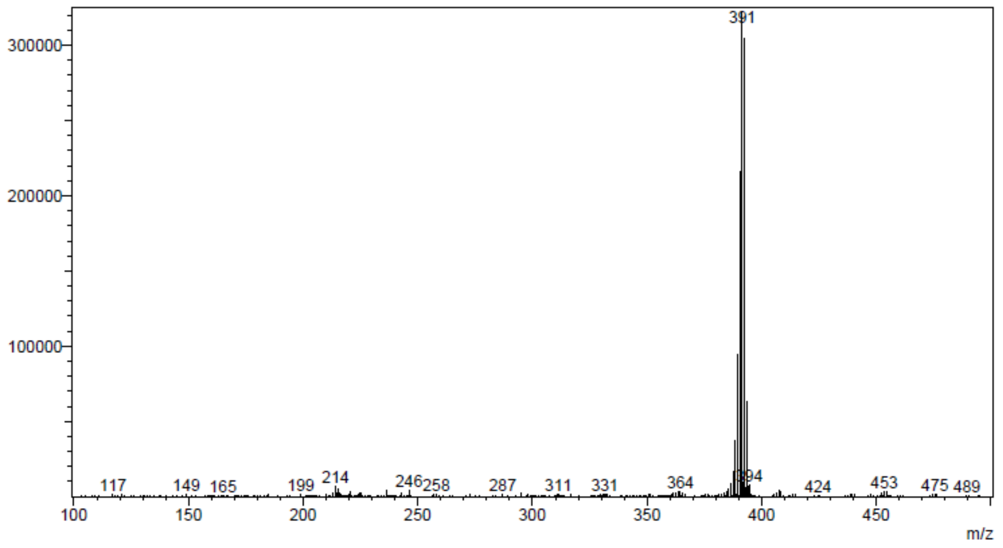

Supplementary Fig. 15. Electrospray ionization mass spectrometry (ESI-MS) analysis of phytantriol-d_{39} [M+Na]+ adduct.

05/05/2025, 11:28 DGet Report for C20H3D39O3

Information

Date:	2025-05-05
Notes:	

Compound Information

Name / ID:	
Formula:	C20H3D39O3
m/z:	369.5582
Adduct:	[M+Na]+
Adduct m/z:	392.5474

Results

Deuteration:	**96.5 %**
Deuteration Ratio Spectra	
D33:	0.75 %
D34:	1.79 %
D35:	3.85 %
D36:	9.69 %
D37:	21.75 %
D38:	34.95 %
D39:	27.22 %

Spectra

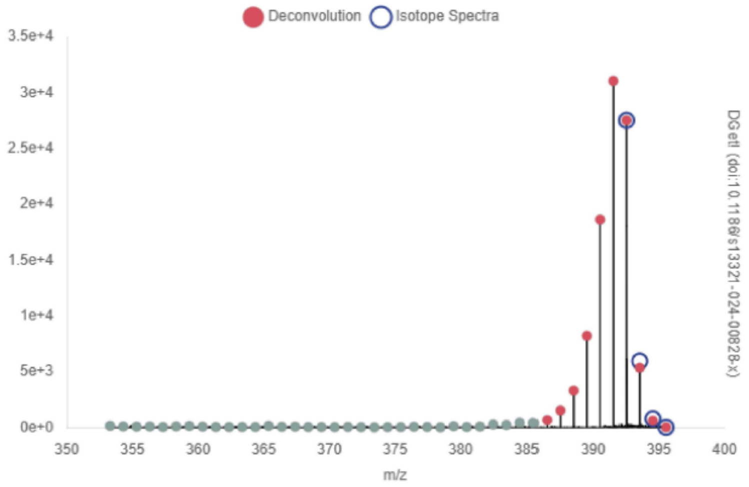

Report generated using the DGet (0.26) web application (0.27.3).

Please cite Lockwood and Angeloski (2024).

For more information see https://github.com/djdt/dget.

Supplementary Fig. 16. Calculation of the average isotopic purity of phytantriol-d_{39} using the distribution of peaks of the [M+Na]+ adduct as shown in Supplementary Fig. 15. The isotopic purity for the molecule was determined as 96.5 ± 2% D. The analysis was performed using the DGet software version 0.26.

Supplementary References

1. Wood, K. et al. Coupling of protein and hydration-water dynamics in biological membranes. *Proc. Natl. Acad. Sci. USA* **104**, 18049–18054 (2007).

2. Salsbury, N. J., Darke, A. & Chapman, D. Deuteron magnetic resonance studies of water associated with phospholipids. *Chem. Phys. Lipids* **8**, 142–151 (1972).

3. Lee, D.-K., Kwon, B. S. & Ramamoorthy, A. Freezing point depression of water in phospholipid membranes: a solid-state NMR study. *Langmuir* **24**, 13598–13604 (2008).

4. Wittebort, R. J., Usha, M. G., Ruben, D. J., Wemmer, D. E. & Pines, A. Observation of molecular reorientation in ice by proton and deuterium magnetic resonance. *J. Am. Chem. Soc.* **110**, 5668–5671 (1988).

5. Sattig, M. et al. NMR studies on the temperature-dependent dynamics of confined water. *Phys. Chem. Chem. Phys.* **16**, 19229–19240 (2014).
