# Supplementary material for: Resolving liquid-to-glass transitions of water under soft nanoconfinement
Source: Nat Commun. 2026 May 8;17:6224. doi: 10.1038/s41467-026-72955-y (PMC13369869; doi:10.1038/s41467-026-72955-y)
Supplement: Supplementary file 2 — Description of Additional Supplementary Information [file 41467_2026_72955_MOESM2_ESM.pdf]

Description of Additional Supplementary Information

Supplementary Data 1

This supplementary data file contains the initial and final configurations from all molecular dynamics simulations performed in this study. Configurations are provided in GROMACS *.gro* format. The data are organized by simulation temperature (190-270 K) and independent run index (1-5). For each run, two coordinate files are included: *initial.gro*, and *final.gro*, corresponding to the initial and final configurations, respectively.
