# Supplementary material for: Resolving liquid-to-glass transitions of water under soft nanoconfinement
Source: Nat Commun. 2026 May 8;17:6224. doi: 10.1038/s41467-026-72955-y (PMC13369869; doi:10.1038/s41467-026-72955-y)
Supplement: Supplementary file 5 — Source Data [file 41467_2026_72955_MOESM5_ESM.pdf]

nature portfolio

Peer Review File

Resolving liquid-to-glass transitions of water under soft nanoconfinement

Corresponding Author: Professor Raffaele Mezzenga

Parts of this Peer Review File have been redacted as indicated to remove third-party material.

This file contains all reviewer reports in order by version, followed by all author rebuttals in order by version.

Attachments originally included by the reviewers as part of their assessment can be found at the end of this file.

Version 0:

Reviewer comments:

Reviewer #1

(Remarks to the Author)

Reviewer #2

(Remarks to the Author)
This work finds that a sub-nanometre layer of water can remain in a glassy state between fluid walls of phytantriol aliphatic alcohol molecules.
A slowing down of water dynamics under soft nano-confinement is observed, occurring within −63 to −20°C across six orders of magnitude in time scales from 10^−6 to 10^−12s,
and a static glass transition ranging between −74 and −64 °C.
The comprehensive study combines SAXS/WAXS, low-temperature microscopy, neutron time-of-flight and backscattering spectroscopy, THz absorption spectroscopy, 2H solid-state NMR,
and atomistic molecular dynamics simulations.

The structural characterization and control experiments are convincing, showing the absence of crystalline water ice in the system of interest,
but its presence in suitably designed control samples, ensuring that water crystalline ice would be detected if present.
The conclusions appear to be fully justified by the experimental and simulation findings. The results constitute an interesting contribution to understanding the glass transition in confinement.
The findings, thus, are of sufficient general interest for publication in Nature Communications. However, major revisions are needed addressing the issues listed below.

The central issue to be addressed consists in the dependence of the neutron spectroscopy results on the momentum transfer q.
The q-dependence of the line broadening in Fig.3e indicates non-Fickian diffusion. The apparent non-zero intersect at q=0 suggests highly confined water mobility.
However, the character of the diffusion process is difficult to judge as the x-axis does not seem to start at zero.
It may be useful to plot the linewidth versus q^2 rather than q to better infer on possible Fickian or Singwii-Sjoelander jump diffusion
or the absence thereof. Moreover, QENS data at higher temperatures should be shown, since the combined range of Emu and Pelican would allow to capture broader Lorentzians.
In this context, the statement on p.6 l. 150: "... and indicating that water explored phase space ergodically on a nanosecond time scale" may be questioned.
What justifies this statement? Water might be mobile but not ergodic, e.g., by being highly confined.

In a similar context, the q-dependence of the elastic fixed-window data should also be shown and discussed (cf. figure 3f).

Obviously, the vibrational part cannot be unambiguously separated from the diffusive part of the scattering contributing to the loss of elastic scattering, but
polynomial approximations of the q-dependence of the elastic intensity, resulting in apparent mean-squared displacements as discussed in existing literature, can be applied.
In this way, the existing data can be analyzed both along T and q and provide a better link to the MD simulations.
Also, the displacement of water molecules on the nanosecond (figure 3) could be associated with their displacement on the microsecond (figure 4).

Would it be possible to calculate scattering functions from the MD simulations to directly compare to the neutron data?

Other points:

Regarding the sample system consisting of "lipidic mesophases of water and an aliphatic alcohol (phytantriol)",
it should be elaborated on the resemblance of phytantriol and lipids. It has to be explained what is meant by "lipidic".

The amorphous structure factor seen in figure 2 a and b also has to be discussed.
Does it correspond to liquid/amorphous water and/or the "lipidic molecules"?
The peaks in figure 2a should also be assigned by a label.

The statement on p.1 l.63/64 "resulting in a strong dependence of the dynamics of water on the confining media" needs a reference.

On p.3 l. 99, the state of "not frozen" may better be termed "not crystalline".

The supplementary figure captions should always state the respective method and instrument used.
Notably, the similarity of THz and neutron vibrational data makes it very difficult to quickly identify the associated method.
In supplementary figure 4 it is unclear which fits are associated.

The lowest q according to the methods section is 0.32 A^-1, but the lowest q data shown in Figure 3 is approx. 0.5 A^-1.

In methods, it should be specified how the sample container contribution to the signal was treated.

Regarding the QENS fits, was the convolution with the resolution function carried out numerically?
A statement should be added, because it will explain the apparent noise on the fits in figure 2.

There seems to be an apparent sloped background in the QENS data (see in particular figure 3d, -105 deg.C). This should be commented on (if space is limited, in the supplementary material).

Extended data figure 4: Maybe explain how the density of states was calculated, since it involves an approximation of the low-q limit.

Reviewer #3

(Remarks to the Author)
The manuscript by Zublin et al. reports on the structure and dynamics of water confined by phytantriol upon cooling in the context of the liquid-to-glass transition of water. The authors use a range of state-of-the-art methods to characterize the samples, including structural characterization using scattering, dynamic characterization using QENS, THz, and NMR methods, and complement their findings with molecular dynamics simulations. The methodology used to characterize these systems is truly impressive, and the manuscript sheds light on all aspects of phase transitions in these samples. I therefore think that this manuscript is highly suitable for publication in Nature Communications. I, however, have a few comments on the interpretation of the data, which should be addressed before I can recommend publication. Details follow below:

- Line 104ff: the authors find evidence for different phases being present (reverse micellar, L_alpha, and L2). However, in the subsequent discussion, there is hardly any discussion of whether some of the observed transitions could also originate from transitions between these phases. It is solely ascribed to water's glass transition. I think this needs to be discussed in more detail. One option to test this might be to add some hydrophobic substance, such as hexane, to expand the hydrophobic phase and do some quick experiments upon cooling. In case the observations are solely due to water, the transitions should be little affected by the hydrophobic substance. I, however, don't know what the phase diagram of this system with hexane looks like, and my suggestion might be a bit naïve.

- Regarding the analysis of the MD simulations. Would it be possible to also analyze the MSD of phytantriol? If the hypothesis that only water undergoes a glass transition, but not phytantriol, is correct, then the MSDs of water and phytantriol should exhibit very different temperature dependences and would strongly support the conclusions.

- I am very skeptical about the volumetric arguments. Firstly, the data in the extended Figure 6 looks too noisy to conclude anything from it. Without error bars (possibly from multiple experiments), I don't see how noise and any phase transition could be distinguished. Secondly (lines 270-272), why does the fluidity of phytantriol imply that temperature-dependent changes can only be due to water? Also, liquids have temperature-dependent densities.

Minor comments
- Line 215, lifetime broadening of vibrational bands is often minor, and I suspect that inhomogeneous broadening and other dephasing mechanisms dominate here. I recommend rephrasing this discussion.
- L223: I suppose there were no deuterium molecules, but D_2O molecules.

Version 1:

Reviewer comments:

Reviewer #1

(Remarks to the Author)
See attached

Reviewer #2

(Remarks to the Author)
The authors have convincingly revised their manuscript in response to my previous comments. In my view, the manuscript is now acceptable for publication in Nature Communications subsequent to very minor additional revisions addressing the items listed below:

The 90% transmission of the samples for neutron spectroscopy given in the Methods section appears to be a rough estimate: "The samples were loaded in annular cans with 0.2 mm gap size ensuring a 90% transmission." It should either be clearly stated that it is an estimate, or the transmission should be calculated.

It may be helpful to quote the temperature protocol from the Methods section in the caption of the new supplementary figure 7, i.e., the fact that the data were recorded upon heating from approximately 4K. This heating ramp rules out any simple freezing-point offset of bulk water as an alternative explanation of the transition seen at approximately -25 deg. C in the MSD, which is, thus, rightly attributed to the Tg of confined water in this system.

Regarding the new statement in the Methods section, "for QENS analysis of the backscattering experiments, the lowest practical Q-value used in the analysis was 0.42 $Å^{-1}$, owing to insufficient counting statistics in the lowest-Q bin at the measured temperatures.": Could it be that at the lowest Q, the energy resolution is also insufficient, since the lowest Q detector might be slightly out of exact backscattering, and therefore have a broader resolution than the higher Q detectors? Also, the effective analyzer surface area seen at lowest Q might be too small, explaining the low signal.

Reviewer #3

(Remarks to the Author)
The authors have addressed all my comments appropriately. I recommend publication of the manuscript in it present form.

Version 2:

Reviewer comments:

Reviewer #1

(Remarks to the Author)
I am perfectly happy now with the clarifications in the revised manuscript.

Reviewer #2

(Remarks to the Author)
By this second revision, the authors have sufficiently accounted for my comments. I recommend publication in Nature Communications.

Open Access This Peer Review File is licensed under a Creative Commons Attribution 4.0 International License, which permits use, sharing, adaptation, distribution and reproduction in any medium or format, as long as you give appropriate credit to the original author(s) and the source, provide a link to the Creative Commons license, and indicate if changes were made.
In cases where reviewers are anonymous, credit should be given to 'Anonymous Referee' and the source.
The images or other third party material in this Peer Review File are included in the article's Creative Commons license, unless indicated otherwise in a credit line to the material. If material is not included in the article's Creative Commons license and your intended use is not permitted by statutory regulation or exceeds the permitted use, you will need to obtain permission directly from the copyright holder.
To view a copy of this license, visit https://creativecommons.org/licenses/by/4.0/

Reviewer #1

We appreciate Reviewers #1's comments on our manuscript. Below is our response with references to the lines of the revised manuscript.

This is an interesting study combining a significant number of measurement techniques with results from careful simulations to elucidate the process of dynamic arrest in water in soft confinement. This provides interesting insight into a regime intermediate between bulk liquid and water confined in fixed nanometer size pores. It is of particular interest to see the diverse ranges in temperature as determined using different techniques, probing the dynamics on different time-scales. Maybe not surprising, but still interesting was to see the intermediate regime with glassy water confined between still liquid tails. The presentation is quite clear and I have very few comments on some aspects where the authors could add some clarification. Otherwise than that, I feel the manuscript is publishable after the minor comments below have been addressed.

1) Line 47. Glassy water in the form of the amorphous LDA and HDA forms do exhibit structural order, but only at short range. Please consider adding "long-range" to describe the structural order of the crystalline phases.

We thank the Reviewer for this helpful suggestion. We have clarified the text in line 47 of the revised manuscript by including the term "long-range" to differentiate crystalline ice as follows:

"The glass transition (T_g) of water identifies the kinetic switch from a supercooled liquid to a glass, where water molecules are kinetically arrested in a disordered configuration[9], unlike in ordered crystalline ice where the solid-state behaviour is associated with long-range structural order."

2) Line 112. It is unclear what "supercoolong effects" are referred to. Please specify since effects of supercooling can manifest in many different properties.

We agree that the original wording was too vague. The sentence has been revised to clarify that we refer specifically to the absence of thermal hysteresis and metastable phase behaviour during the cooling-heating cycle. We have modified the sentence in line 115 of the revised manuscript as follows:

"The phase behaviour was fully reversible upon heating, with no observable thermal hysteresis or metastable phases that would indicate supercooling-related effects."

3) Lines 149-150. The measurements show an enhanced mobility of H2O over D2O, but the jump to "ergodically" exploring the phase space seems rather large since this would (formally) require proof that "all" possible configurations are explored on the relevant time scale.

We thank the Reviewer for highlighting this issue and agree that the original statement went beyond what could be supported. We have deleted the relevant part of the sentence in line 154, which now reads as follows:

"At −5 °C, the QENS spectrum of the mesophase with H_2O was significantly broader than that of the mesophase containing D_2O (Fig. 3b), consistent with an enhanced mobility of H_2O molecules ~~and indicating that water explored phase space ergodically~~ on a nanosecond time scale."

4) Line 289. The "rate-dependence" needs to be specified. The rate of change in temperature seems to have been constant. I assume the authors mean the time-scale of the measurements, but it would be good to clarify.

We agree that the original wording was ambiguous. Our intention was to refer to the dependence of the apparent glass transition temperature on the dynamical time scale probed by each technique, rather than on the temperature ramp rate, which was kept constant at one degree per minute in the experiments. We have revised the text in line 302 of the revised manuscript to explicitly refer to the observation time scale of the measurements as follows:

"Our results indicate that, as expected, the exact location of the liquid-to-glass transition depends on the dynamical time scale probed by the measurement technique."

Reviewer #2

This work finds that a sub-nanometre layer of water can remain in a glassy state between fluid walls of phytantriol aliphatic alcohol molecules.
A slowing down of water dynamics under soft nano-confinement is observed, occurring within −63 to −20°C across six orders of magnitude in time scales from 10^−6 to 10^−12s, and a static glass transition ranging between −74 and −64 °C.
The comprehensive study combines SAXS/WAXS, low-temperature microscopy, neutron time-of-flight and backscattering spectroscopy, THz absorption spectroscopy, 2H solid-state NMR, and atomistic molecular dynamics simulations.

The structural characterization and control experiments are convincing, showing the absence of crystalline water ice in the system of interest, but its presence in suitably designed control samples, ensuring that water crystalline ice would be detected if present.
The conclusions appear to be fully justified by the experimental and simulation findings. The results constitute an interesting contribution to understanding the glass transition in confinement.
The findings, thus, are of sufficient general interest for publication in Nature Communications.
However, major revisions are needed addressing the issues listed below.

We appreciate Reviewers #2's comments on our manuscript. Below is our response with references to the lines of the revised manuscript.

The central issue to be addressed consists in the dependence of the neutron spectroscopy results on the momentum transfer q.
The q-dependence of the line broadening in Fig.3e indicates non-Fickian diffusion. The apparent non-zero intersect at q=0 suggests highly confined water mobility.
However, the character of the diffusion process is difficult to judge as the x-axis does not seem to start at zero.
It may be useful to plot the linewidth versus q^2 rather than q to better infer on possible Fickian or Singwii-Sjoelander jump diffusion or the absence thereof.

We thank the Reviewer for this valuable suggestion. We have redrawn the plot in Figure 3e on page 7 of the revised manuscript to show the linewidth vs. Q^2 and to include zero on the x-axis as follows:

e

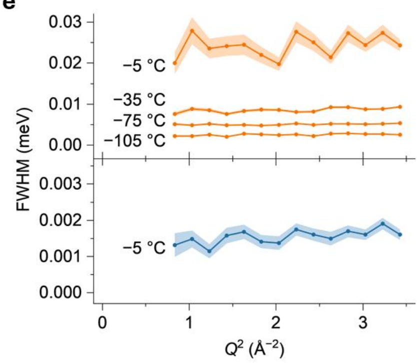

We have adapted the respective Figure caption to include *"…as a function of Q^2."*.

In the revised manuscript (line 173ff), we have strengthened the wording to more accurately describe the localised nature of the dynamics inferred from the Q-independent linewidth. In particular, the revised text now explicitly states that no evidence for Fickian or Singwi–Sjölander jump diffusion is

observed within the experimental length-scale window, and includes a reference to the 1960 Singwi and Sjölander article (https://doi.org/10.1103/PhysRev.119.863):

"The full-width at half-maximum (FWHM) of the Lorentzian as a function of Q provides information on the time and length scales of the dynamics[32]. The Q-independent linewidths indicate localised dynamics for phytantriol and water (Fig. 3e), with no evidence for Fickian or Singwi–Sjölander jump diffusion[35] down to the maximum probed length scale of ~15 Å (Q = 0.42 Å^{-1})."

Moreover, QENS data at higher temperatures should be shown, since the combined range of Emu and Pelican would allow to capture broader Lorentzians.

We thank the Reviewer for this suggestion. While the Reviewer's suggestion is certainly intriguing, we would like to emphasize that the focus of the submitted manuscript lies on dynamics in the temperature range below 0 °C. Due to the limited and highly competitive beamtime available on Emu and Pelican, we restricted our data collection to temperatures of -5 °C and below.

The Reviewer is correct that the combined dynamic range of Emu and Pelican would, in principle, allow access to broader Lorentzians. However, in the present case the time scale of Pelican is too fast to capture the diffusive dynamics of confined water that are relevant to identifying its glass transition. Consequently, an analysis analogous to that performed using the Emu QENS data, where we could separate the dynamics from water and phytantriol, is not feasible.

For this reason, the Pelican QENS data do not provide additional constraints on the glass transition of water and are therefore not included in the manuscript, except for the low-temperature measurements at -75 °C and -135 °C, which are used to identify and confirm the boson peak.

In this context, the statement on p.6 l. 150: "... and indicating that water explored phase space ergodically on a nanosecond time scale" may be questioned.
What justifies this statement? Water might be mobile but not ergodic, e.g., by being highly confined.

We thank the Reviewer for pointing out this issue and agree that the original sentence was too speculative. The relevant part of the sentence in line 154 has been removed in the revised manuscript, which now reads as follows:

"At −5 °C, the QENS spectrum of the mesophase with H_2O was significantly broader than that of the mesophase containing D_2O (Fig. 3b), consistent with an enhanced mobility of H_2O molecules ~~and indicating that water explored phase space ergodically~~ on a nanosecond time scale."

In a similar context, the q-dependence of the elastic fixed-window data should also be shown and discussed (cf. figure 3f).
Obviously, the vibrational part cannot be unambiguously separated from the diffusive part of the scattering contributing to the loss of elastic scattering, but polynomial approximations of the q-dependence of the elastic intensity, resulting in apparent mean-squared displacements as discussed in existing literature, can be applied.
In this way, the existing data can be analyzed both along T and q and provide a better link to the MD simulations.

Also, the displacement of water molecules on the nanosecond (figure 3) could be associated with their displacement on the microsecond (figure 4).

We thank the Reviewer for the valuable comment. In order to show the Q-dependence of the elastic fixed window data, we have calculated the MSDs using both the Gaussian and the polynomial approximation. The apparent mean-squared displacements, as derived from the elastic intensity data shown in Figure 3g of the manuscript, compare as follows:

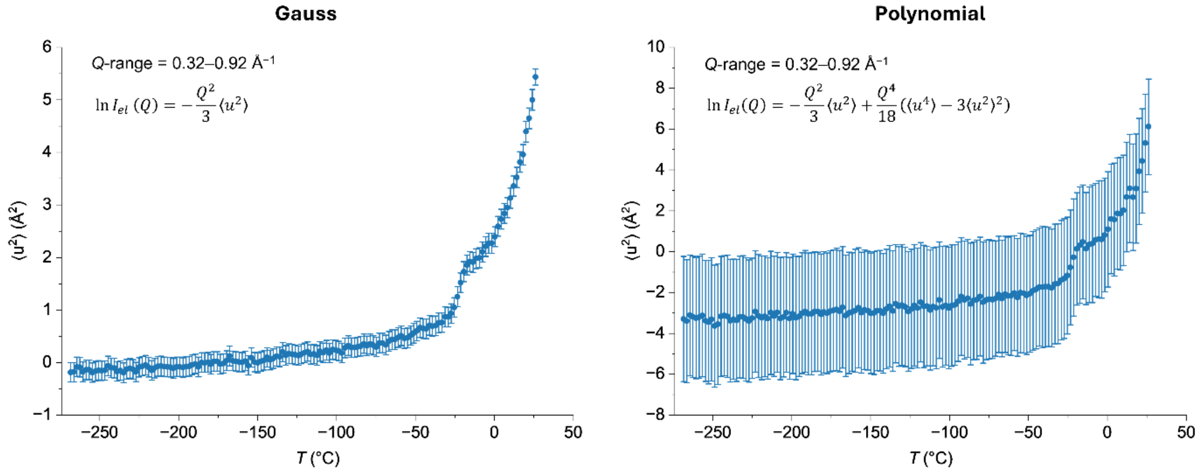

The resulting MSD vs. T plots demonstrate that the polynomial expansion does not describe the data accurately in the low-temperature regime. In particular, higher-order cumulant fits are known to become unreliable in the low-temperature, small-displacement limit, where the elastic intensity deviates only weakly from unity and the contribution of the higher-order terms cannot be robustly constrained. Also, the associated error of the polynomial MSD is significantly larger than in the Gaussian approximation, which reflects the strong correlation between the second- and fourth-order cumulants within the Q-range considered for the analysis and the small-displacement regime of the present data.

This limitation of the polynomial expansion has been discussed in the context of Debye-Waller and extended X-ray absorption fine structure (EXAFS) analyses, where the convergence and reliability of cumulant coefficients depend critically on the data range and the magnitude of disorder[a,b]. Moreover, it has been reported that even in heterogeneous systems the Gaussian approximation can reproduce qualitatively similar trends compared to models explicitly accounting for dynamical heterogeneity[c]. As consequence, the Gaussian approximation provides a more stable and statistically reliable estimate of the MSD in our case and is therefore presented in the revised manuscript.

Most importantly with respect to the main point of the glass transitions discussed in the manuscript—and irrespectively of whether Gaussian or polynomial fits are used to fit the MSD data—the glass transition of water is clearly visible in the temperature range at approximately -25 °C. We have added the Gaussian MSD plot to the revised Supplementary Information on page 5 to show the trend in change of the MSD with temperature:

“

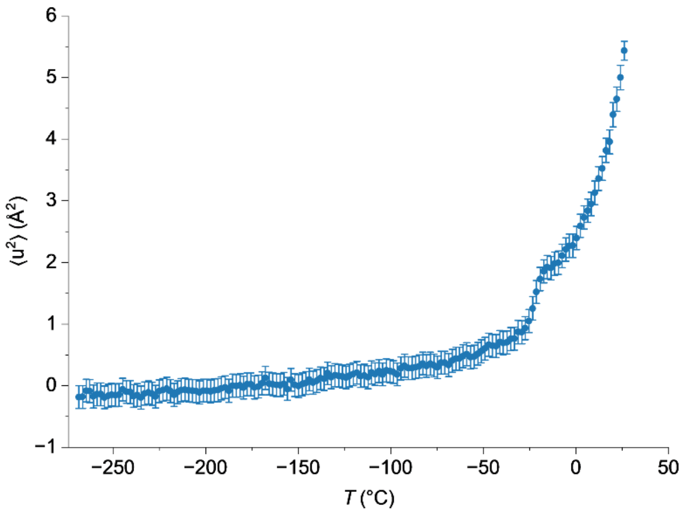

Supplementary Fig. 7. *Apparent mean-squared displacement* $\langle u^2 \rangle$ *vs. temperature of 6.7 wt% H_2O in phytantriol-d_{39} derived from the elastic FWS data shown in Fig. 3g. The T_g of water is evident at around −25 °C by a sudden change in the slope of the mean-squared displacement. The Q-range considered for the analysis was 0.32–0.92 Å^{-1}.*

”

In the revised manuscript, we have added a statement in line 179 and refer to Supplementary Fig. 7:

“This observation is further supported by a pronounced increase in the apparent mean-squared displacement extracted from the Q-dependence of the elastic intensity at low Q (ref. 32) (Supplementary Fig. 7).”

We have added a section to Methods starting at line 389 in the revised manuscript describing how the MSD values were extracted:

“The apparent mean-squared displacement (MSD) was obtained from the Q-dependence of the elastic FWS data using a Gaussian approximation:

$$\ln I_{el}\,(Q) = -\frac{Q^2}{3}\langle u^2 \rangle \qquad (2)$$

where I_{el} is the elastic intensity and $\langle u^2 \rangle$ the MSD. The Q-range considered for the analysis was 0.32–0.92 Å^{-1} and 150 temperature points between −269 and 27 °C were evaluated. The uncertainty of the MSD was derived from the standard error of the fitted slope.”

Finally, we appreciate the Reviewer’s insightful suggestion to relate the neutron-derived MSD on the nanosecond to the displacements obtained from MD simulations on the microsecond. However, the MSD obtained from elastic neutron scattering represents the incoherent average over all hydrogen atoms in the sample and therefore reflects the combined dynamics of both water and phytantriol. Although the use of deuterated phytantriol-*d_{39}* increases the relative incoherent contribution of H_2O

from 7% to 42%, the elastic signal remains significantly influenced by hydrogen atoms in the phytantriol headgroups. In contrast, MD simulations allow selective extraction of the MSD of water molecules only. Consequently, it is not straightforward to make a direct comparison between the neutron-derived MSD and the MSD obtained from simulations, since the experimental observable inherently reflects a composite dynamical signal.

a. Dalba, G., Fornasini, P. & Rocca, F. Cumulant analysis of the extended x-ray-absorption fine structure of β-AgI. *Phys. Rev. B* **47**, 8502–8514 (1993). https://doi.org/10.1103/PhysRevB.47.8502
b. Fornasini, P., Monti, F. & Sanson, A. On the cumulant analysis of EXAFS in crystalline solids. *J. Synchrotron Radiat.* **8**, 1214–1220 (2001). https://doi.org/10.1107/s0909049501014923
c. Zeller, D., Telling, M. T. F., Zamponi, M., García Sakai, V. & Peters, J. Analysis of elastic incoherent neutron scattering data beyond the Gaussian approximation. *J. Chem. Phys.* **149**, (2018). https://doi.org/10.1063/1.5049938

Would it be possible to calculate scattering functions from the MD simulations to directly compare to the neutron data?

We thank the Reviewer for this suggestion. In principle, one can compute the theoretical scattering function S(Q,ω) from MD trajectories by double Fourier transforming the appropriate time-correlation functions, and one of the co-authors of the present submitted manuscript has been involved in a similar effort carried out in condensed matter systems (https://doi.org/10.1016/j.actamat.2021.117026). However, for the present lipid–water mesophase this route is not practically feasible. While in principle MD covers the time scales probed by QENS (from pico- to micro-seconds), due to storage constraints, we have saved the trajectory with a time interval between successive configurations of 300 ps. This prevents an accurate determination of the QENS spectra from the simulation data in the region of frequencies where experiments have been performed. Additionally, the structural and dynamical heterogeneity of the system (e.g., mixed coherent and incoherent scattering from lipids and water, different head/tail scattering densities, complex overlapping motions) renders the decomposition of the simulated S(Q,ω) into physically interpretable contributions ambiguous and impractical. For these reasons, while the suggestion is highly intriguing, we prefer not to pursue this comparison.

Other points:

Regarding the sample system consisting of "lipidic mesophases of water and an aliphatic alcohol (phytantriol)", it should be elaborated on the resemblance of phytantriol and lipids. It has to be explained what is meant by "lipidic".

We thank the Reviewer for the suggestion, which will make the manuscript more accessible to a broader audience. We have clarified in the revised manuscript that phytantriol, although not a lipid in the strict chemical sense, is an amphiphilic molecule that self-assembles with water into mesophases analogous to those formed by lipid-water systems. The term “lipidic mesophase” is therefore used to describe the lipid-like self-assembled nanostructures rather than the specific chemical identity of the amphiphilic molecule.

We have added the following section to Methods starting at line 329 in the revised manuscript to address the resemblance between phytantriol and lipids, including a reference[d] to a standard work on phytantriol-water mesophases:

"Phytantriol is an amphiphilic aliphatic alcohol that self-assembles with water into well-defined lyotropic mesophases[55], analogous to those formed by lipid-water systems. Although phytantriol is not a lipid in the strict chemical sense, its molecular architecture and phase behaviour closely resemble those of lipid-based amphiphiles. The term 'lipidic mesophase' is used to describe these lipid-like self-assembled nanostructures."

In line 76 of the revised manuscript, we added a reference to the Methods section:

"We resolve the slowing down of the dynamics of soft nanoconfined water across six orders of magnitude in time scales, from 10^{-6} to 10^{-12} s, by an unprecedented combination of dynamic (quasi-elastic neutron scattering, synchrotron THz absorption spectroscopy and solid-state NMR), static (X-rays scattering) and in silico (atomistic molecular dynamic simulations) methods, applied to self-assembled lipidic mesophases of water and an aliphatic alcohol (phytantriol, see Methods) as a model system for soft nanoconfinement."

We have replaced the term "lipidic" with "soft" in the title of the revised manuscript, which now reads:

"Resolving the liquid-to-glass transition(s) of water under soft nanoconfinement"

d. Barauskas, J. & Landh, T. Phase behavior of the phytantriol/water system. *Langmuir* **19**, 9562–9565 (2003). https://doi.org/10.1021/la0350812

The amorphous structure factor seen in figure 2 a and b also has to be discussed.
Does it correspond to liquid/amorphous water and/or the "lipidic molecules"?
The peaks in figure 2a should also be assigned by a label.

We thank the Reviewer for this comment and would like to reply by first giving a brief explanation followed by how we clarified this issue in the revised manuscript.

The broad structure factor peak originates from the packing of the phytantriol (lipidic) molecules and allows to distinguish polymorphism in lamellar phases. In our data, the absence of Bragg peaks in the structure factor is characteristic of the fluid lamellar phase (L_α), in which the lipidic molecules self-assemble into lamellar membranes with disordered packing, in contrast to the ordered gel-like (denoted as L_β in the literature) and crystalline (L_c) lamellar phases. Upon cooling from 20 to −120 °C, the structure factor peak shifts toward higher Q-values, whereas the lamellar Bragg peaks of the L_α phase shift toward lower Q-values. These observations are not contradictory: the former reflects tighter local packing of phytantriol molecules due to reduced thermal motion, while the latter reflects an increase in the lamellar repeat distance (as can be seen in Fig. 2c, top panel), as stiffened bilayers move farther apart despite an unchanged hydration level.

To address the point raised by the Reviewer, we have modified the text starting at line 108 in the revised manuscript as follows:

"Upon cooling 7.5 wt% H_2O in phytantriol from 20 to −120 °C, the symmetry shifted from a mixed reverse micellar and lamellar ($L_2 + L_\alpha$) to a highly ordered lamellar phase (L_α). The L_α phase remained

fluid and non-crystalline down to −120 °C due to the non-freezing properties of phytantriol[29], which is supported by a broad structure factor peak centred at Q ~1.3 Å^{-1} in Figure 2a. As a control, Supplementary Fig. 1b shows a lamellar crystalline ~~structure~~ phase formed in a monoolein-based mesophase of identical composition, which rapidly froze between −5 and −10 °C. In the case of phytantriol, the lattice size slightly increased due to tighter lateral packing associated with reduced thermal mobility upon cooling before reaching a constant value of ~34–35 Å below −60 °C (Fig. 2c, top panel).”

In the revised manuscript, we have labelled the Bragg peaks in Figure 2a consecutively from 1 to 5, corresponding to the first five orders of the L_α phase, and have added a description in the figure caption in line 132:

"

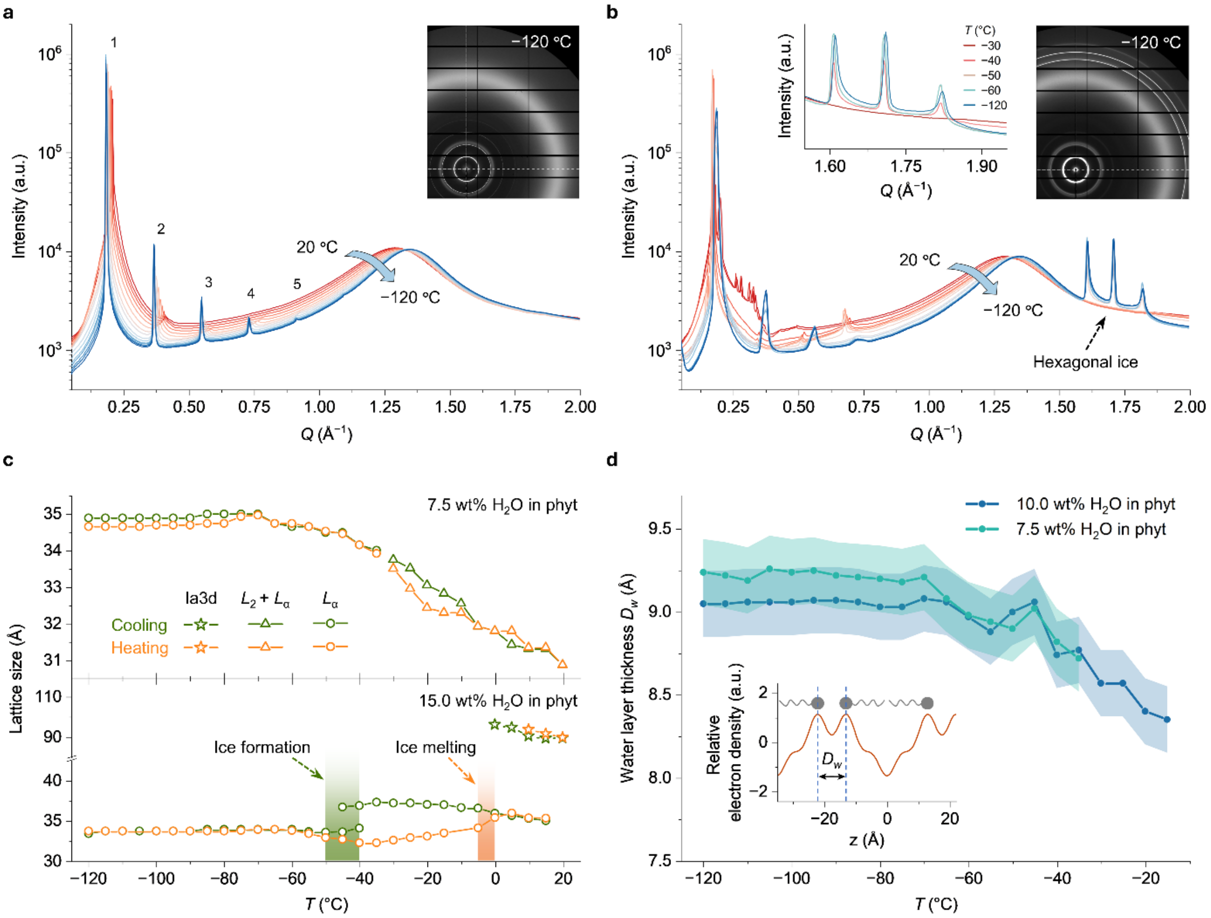

***Fig. 2. Structural characterisation of non-freezing lipidic mesophase using SAXS/WAXS. a,b**, Selection of SAXS/WAXS profiles of 7.5 wt% (**a**) and 15.0 wt% H_2O in phytantriol (**b**) during cooling from 20 to −120 °C in 10 °C increments. The cooling rate was 1 °C min^{-1}. <u>The first five Bragg reflections (low-Q to higher-Q order) of the L_α phase are labelled in **a**</u>. Top-right insets show a 2D-detector image taken at −120 °C. Top-centre inset in **b** shows a zoom of hexagonal ice Bragg reflections. **c**, Lattice size and phase symmetry (Ia3d, L_2 + L_α, L_α) for 7.5 and 15.0 wt% H_2O in phytantriol as a function of temperature during the cooling and heating cycle derived from Bragg reflections as shown in **a** and **b** and described in Methods. **d**, Examples of the water layer thickness (D_w) as a function of temperature in the pure lamellar phase (L_α) of 7.5 and 10.0 wt% H_2O in phytantriol, obtained from the EDP. The error bands denote the uncertainty based on the instrument resolution. Inset, representative location of the water layer (blue dashed lines) between lipidic headgroups in the EDP. phyt, phytantriol; a.u., arbitrary units.*

"

The statement on p.1 l.63/64 "resulting in a strong dependence of the dynamics of water on the confining media" needs a reference.

We have added a reference to Cerveny *et al.* (2016) (https://doi.org/10.1021/acs.chemrev.5b00609) in line 65 of the revised manuscript and thank the Reviewer for pointing this out.

On p.3 l. 99, the state of "not frozen" may better be termed "not crystalline".

We agree with the Reviewer and have changed the respective sentence in line 100 of the revised manuscript:

"The absence of characteristic hexagonal ice reflections[28] at Q = 1.61, 1.71 and 1.82 $Å^{-1}$ revealed that H_2O is not crystalline."

Consequently, we have replaced the term "frozen" with "crystalline" in line 239 of the revised manuscript:

"At −60 °C, the signal was recovered and the C_Q-value converged to that of crystalline D_2O (C_Q = 198 kHz, horizontal grey dashed line in Fig. 4c),…"

Additionally, we have adapted the wording from "non-frozen" to "non-freezing" in line 127 of the revised manuscript to more accurately describe the property of the confined water:

"Thus, using SAXS/WAXS, we demonstrated the consistent feature of phytantriol-based mesophases to provide sub-nanometre layers of non-freezing water confined between lipidic bilayers, identifying the static and dynamics regimes of interest in this study."

The supplementary figure captions should always state the respective method and instrument used.
Notably, the similarity of THz and neutron vibrational data makes it very difficult to quickly identify the associated method.
In supplementary figure 4 it is unclear which fits are associated.

We acknowledge the lack of clarity in the captions of the Supplementary Figures and thank the Reviewer for this comment. We have addressed this in the revised Supplementary Information by indicating the name of the technique at the beginning of each caption. In particular, for Supplementary Fig. 9 in the revised manuscript (originally Supplementary Fig. 4), we have clarified the associated fit. It now states on page 7 in the revised Supplementary Information:

"Supplementary Fig. 9. THz Absorption Spectroscopy. *Residual errors from the spectral fit of the full THz signal, as presented in the Methods section in Eq. (6), at all investigated temperatures for 10.0 wt% H_2O in phytantriol. These residuals reflect the etalon effects present in the experimental absorption spectra."*

We have also added the respective method and instrument used in Supplementary Figures 3, 5, 6, 8, 10 and 11.

The lowest q according to the methods section is 0.32 A^-1, but the lowest q data shown in Figure 3 is approx. 0.5 A^-1.

We apologise for the confusion created by not using clear spelling and thank the Reviewer for highlighting this issue. The Reviewer is correct in that the lowest accessible Q by the backscattering instrument was 0.32 A^-1, as stated in Methods. However, the lowest practically used Q in the QENS analysis was 0.42 A^-1, as Q-values below that resulted in very noisy signal and poor fitting stability. For the elastic FWS, we were able to use the full Q-range of 0.32–1.72 A^-1.

In the revised manuscript, we have addressed this point by modifying the Methods section in line 380 as follows:

"For QENS analysis of the backscattering experiments, the lowest practical Q-value used in the analysis was 0.42 Å^{-1}, owing to insufficient counting statistics in the lowest-Q bin at the measured temperatures. Data were binned in increments of 0.1 Å^{-1} in a Q-range of 0.42–1.72 Å^{-1} and fitted with a Lorentzian for each quasi-elastic contribution."

In line 176 of the revised manuscript, we have changed the text as follows:

"The Q-independent linewidths indicate localised dynamics for phytantriol and water (Fig. 3e), with no evidence for Fickian or Singwi–Sjölander jump diffusion[35] down to the maximum ~~accessible~~ probed length scale of ~15 Å (Q = 0.42 Å^{-1})."

In methods, it should be specified how the sample container contribution to the signal was treated.

We have clarified how the signal of the empty can was treated in the methods (line 378) of the revised manuscript by adding the following sentence:

"Scattering from an empty sample, measured under the same instrumental configuration and temperature conditions as the samples, was subtracted as background."

Regarding the QENS fits, was the convolution with the resolution function carried out numerically? A statement should be added, because it will explain the apparent noise on the fits in figure 2.

The Reviewer is correct and the convolution was done numerically. In response to this comment, we have revised the relevant section in Methods (line 378) to provide further clarification regarding the numerical convolution fitting in Figure 3 (and Supplementary Fig. 5):

"For QENS analysis of the backscattering experiments, the lowest practical Q-value used in the analysis was 0.42 Å^{-1}, owing to insufficient counting statistics in the lowest-Q bin at the measured temperatures. Data were binned in increments of 0.1 Å^{-1} in a Q-range of 0.42–1.72 Å^{-1} and fitted with a Lorentzian for each quasi-elastic contribution. Data were numerically convoluted with the instrument resolution[32], leading to small point-to-point fluctuations in the fitted curves that do not affect the extracted linewidths."

There seems to be an apparent sloped background in the QENS data (see in particular figure 3d, -105 deg.C). This should be commented on (if space is limited, in the supplementary material).

We thank the Reviewer for this observation. The apparent asymmetry and sloped background visible in the QENS spectra in in Figure 3d and Supplementary Fig. 5 originate from the intrinsic asymmetry of the instrument resolution function of the Emu backscattering spectrometer. For this reason, all numerical convolution fits were performed using an experimentally measured resolution function.

Such resolution asymmetry is not uncommon in backscattering QENS instruments. As examples, QENS data collected on the BASIS spectrometer at the Oak Ridge National Laboratory (USA) also exhibit asymmetric peak profiles, which are treated explicitly via resolution convolution (https://doi.org/10.1021/acs.jpcc.9b01873, Figure 1; https://doi.org/10.1039/D4RA00565A, Figure 3).

To clarify this point, we have added the following sentence in Methods (line 385) of the revised manuscript:

"The Emu backscattering spectrometer exhibits a known resolution asymmetry, which is reflected in the raw spectra and accounted for in the convolution."

Extended data figure 4: Maybe explain how the density of states was calculated, since it involves an approximation of the low-q limit.

We thank the reviewer for this helpful suggestion. We have now added a description in the Methods section starting at line 394 in the revised manuscript explaining how the reduced vibrational density of states $g(\omega)/\omega^2$ (Supplementary Fig. 6 in the revised Supplementary Information) was calculated:

"The reduced vibrational density of states ($g(\omega)/\omega^2$) was derived from the time-of-flight data using the ComputeIncoherentDOS algorithm implemented in Mantid. In brief, $S(Q,\omega)$ is transformed to the generalised density of states within the incoherent approximation using:

$$g(Q,\omega) = \frac{\hbar\omega}{Q^2} S(Q,\omega)\left(1 - e^{\frac{-\hbar\omega}{kT}}\right) \quad (3)$$

where the scattering function $S(Q,\omega)$ is corrected for temperature-dependent phonon populations using the Bose factor at energy $E = \hbar\omega$. We then integrate $g(Q,\omega)$ over the measured Q-range for each energy. Finally, the spectrum is weighted by ω^2, which yields $g(\omega)/\omega^2$ to emphasise the boson peak."

Reviewer #3

The manuscript by Zublin et al. reports on the structure and dynamics of water confined by phytantriol upon cooling in the context of the liquid-to-glass transition of water. The authors use a range of state-of-the-art methods to characterize the samples, including structural characterization using scattering, dynamic characterization using QENS, THz, and NMR methods, and complement their findings with molecular dynamics simulations. The methodology used to characterize these systems is truly impressive, and the manuscript sheds light on all aspects of phase transitions in these samples. I therefore think that this manuscript is highly suitable for publication in Nature Communications. I, however, have a few comments on the interpretation of the data, which should be addressed before I can recommend publication. Details follow below:

We appreciate Reviewers #3's comments on our manuscript. We thank the Reviewer for the positive assessment and for acknowledging the value of our methodological approach.

Below is our response with references to the lines of the revised manuscript.

- Line 104ff: the authors find evidence for different phases being present (reverse micellar, L_alpha, and L2). However, in the subsequent discussion, there is hardly any discussion of whether some of the observed transitions could also originate from transitions between these phases. It is solely ascribed to water's glass transition. I think this needs to be discussed in more detail. One option to test this might be to add some hydrophobic substance, such as hexane, to expand the hydrophobic phase and do some quick experiments upon cooling. In case the observations are solely due to water, the transitions should be little affected by the hydrophobic substance. I, however, don't know what the phase diagram of this system with hexane looks like, and my suggestion might be a bit naïve.

We agree with the Reviewer that an experimental approach in which a hydrophobic substance such as hexane is added to change the phase behaviour would be ideal, in theory. However, adding a third, hydrophobic component with different chemical potential to a binary system of phytantriol-water commonly results in highly altered phase diagrams, as previous literature have summarised for different lipidic mesophase systems[e,f]. For phytantriol, we have unpublished data showing the influence of small molecules, such as hexane, on a binary phytantriol-water system, by inducing more curvature, which promotes e.g. hexagonal phases and drives the system into phase separation even at low percentages of added hexane.

Instead, it is possible to reply purely on thermodynamic grounds: we observe a single dominant relaxation process of water that spans tens of degrees from the coexistence into the single phase regime, inferring that the water Tg is decoupled from the order-order transition (OOT), otherwise a splitting of the relaxation time would be observed in correspondence of the OOT. In strong support of this picture, we have previously shown[g] that water dynamics are largely independent of topology.

We have addressed this point in the revised manuscript as follows:

In line 108, we introduce the term "OOT":

"Upon cooling 7.5 wt% H_2O in phytantriol from 20 to −120 °C, the symmetry shifted from a mixed reverse micellar and lamellar (L_2 + L_α) to a highly ordered lamellar phase (L_α) via an order-order transition (OOT)."

In lines 309–312 of the revised manuscript, we have added a sentence to emphasise on the decoupling of the water Tg from the OOT including a reference[d]. The modified section now reads:

"Particularly noteworthy is the observation that, while all glassy regimes identified here remain above the previously reported range for soft nanoconfined water, techniques probing comparable time scales (MD simulations and ^{2}H ssNMR) yield overlapping temperature ranges. Remarkably, we find a narrow window between −31 and −35 °C that is compatible with a dynamic glass transition of water probed across six orders of magnitude in observation time scales by THz absorption spectroscopy, neutron scattering and ^{2}H ssNMR. Importantly, all three experimental dynamical techniques reveal a single dominant relaxation process for water within the identified T_g range, which extends even above the OOT temperature (≥−30 °C), inferring that water dynamics are not governed by changes in the confining symmetry, consistent with our earlier experimental, numerical and theoretical findings[53]. Finally, static techniques such as SAXS yield a relatively lower temperature range of the liquid-to-glass transition (yet above the previously reported range for the T_g of soft nanoconfined water), as it should be expected for infinitely slow measurements of the T_g."

We have added a horizontal dashed line in Figure 4h on page 9 of the revised manuscript to facilitate direct comparison between the temperature range associated with the water Tg areas and the coexistence regime limited by the order-order transition (OOT) temperature, thereby indicating their partial overlap and supporting the discussion included in the revised main text:

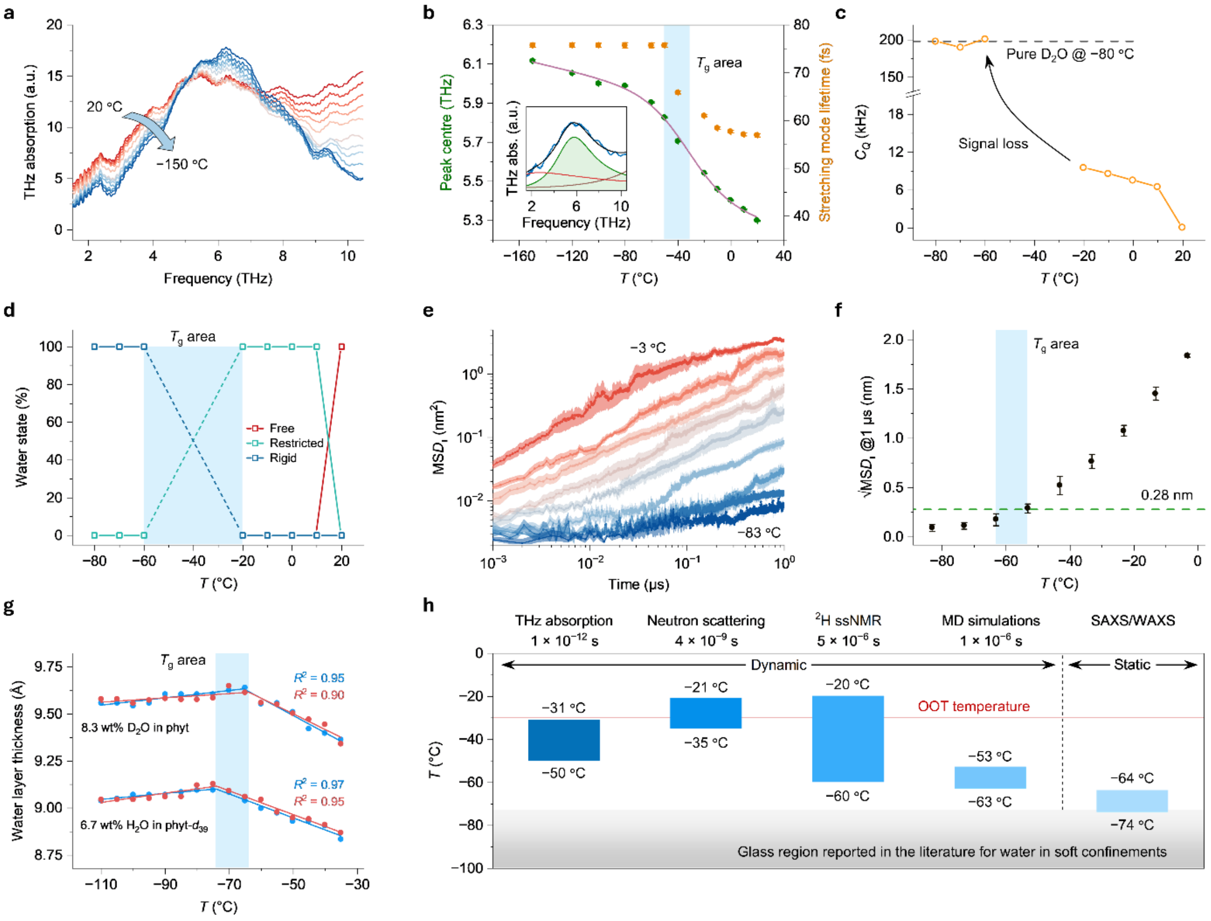

The Figure caption in lines 261–263 of the revised manuscript is modified as follows:

"

***h**, Overview of the T_g areas (in blue) determined in this work by various techniques. The T_g range of water in different soft confinements reported in the literature[19] is indicated ~~in~~ by the grey area. The OOT temperature at −30 °C, below which the system exists as a single phase, is represented as a solid red line.*

"

e. Caboi, F. et al. Addition of hydrophilic and lipophilic compounds of biological relevance to the monoolein/water system. I. Phase behavior. *Chem. Phys. Lipids* **109**, 47–62 (2001). https://doi.org/10.1016/S0009-3084(00)00200-0

f. Huang, Y. & Gui, S. Factors affecting the structure of lyotropic liquid crystals and the correlation between structure and drug diffusion. *RSC Adv.* **8**, 6978–6987 (2018). https://doi.org/10.1039/C7RA12008G

g. Zunzunegui-Bru, E. et al. Universality in the Structure and Dynamics of Water under Lipidic Mesophase Soft Nanoconfinement. *ACS Nano* **18**, 21376–21387 (2024). https://doi.org/10.1021/acsnano.4c05857

- Regarding the analysis of the MD simulations. Would it be possible to also analyze the MSD of phytantriol? If the hypothesis that only water undergoes a glass transition, but not phytantriol, is correct, then the MSDs of water and phytantriol should exhibit very different temperature dependences and would strongly support the conclusions.

We are grateful for this meaningful comment from the reviewer. A comparison of the mean square displacement (MSD) of water and phytantriol would indeed provide valuable information. However, due to the significantly slower motion of lipids, approximately at least two orders of magnitude slower than that of water (Fahey, P. F., and W. W. Webb, *Biochemistry* 17, 3046–3053, 1978. https://doi.org/10.1021/bi00608a016), obtaining comparable dynamical results for water and phytantriol would require simulation times on the order of 100 microseconds. Given that our available computational resources allow approximately 0.5 microseconds per day, this would require more than six months of continuous simulation for a single run. Furthermore, the combined experimental results from neutron scattering and microscopy shown in Fig. 3 indicate that phytantriol exhibits glassy behaviour only below approximately −105 °C. We acknowledge that this point may not have been clearly conveyed and thank the reviewer for this valuable insight. We have therefore strengthened this message in the manuscript's conclusion. The corrected main text in lines 295–299 of the revised manuscript now reads:

"Here, we report the observation of a glassy layer of water as thin as 0.8–0.9 nm (~3 water molecules) confined between liquid lipidic walls. This suggests that the relaxation processes of water are decoupled from those of the phytantriol hydrophobic tails, which, based on the combined macroscopic behaviour of the mesophase (Fig. 3a) and neutron scattering (Fig. 3d), exhibit signatures of glassy behaviour only at temperatures below −105 °C."

In addition, we have expanded the discussion to explicitly compare the Tg values obtained in the present work with earlier results from our group based on differential scanning calorimetry and broadband dielectric spectroscopy. This clarification strengthens the central conclusion that the Tg of

water under soft lipid nanoconfinement is higher than previously reported. In line 286 of the revised manuscript, we have modified the sentence to:

"Our values for the liquid-to-glass transition(s) of water under soft lipidic nanoconfinement are found systematically above the previously reported range for the T_g of water in soft nanoconfinement (Fig. 4h)*, including earlier values reported by some of us for the same system based on differential scanning calorimetry and broadband dielectric spectroscopy*[29]."

- I am very skeptical about the volumetric arguments. Firstly, the data in the extended Figure 6 looks too noisy to conclude anything from it. Without error bars (possibly from multiple experiments), I don't see how noise and any phase transition could be distinguished. Secondly (lines 270-272), why does the fluidity of phytantriol imply that temperature-dependent changes can only be due to water? Also, liquids have temperature-dependent densities.

Here, we need first of all to apologize because our poor language in the earlier submission must certainly have misled the Reviewer, and we are sorry about this. The relevant figure is actually Figure 4g and not Extended Data Figure 6, which we originally wanted to use only in support of Figure 4g, but which is actually introducing misunderstanding, and has therefore been removed from the revised manuscript.

Figure 4g shows water layer thickness vs. temperature and given the 2D symmetry, this corresponds to volume of water vs. temperature. According to Ehrenfest classification of phase transitions, a phase transition is of the first order (e.g. melting) if the Gibbs free energy exhibits a discontinuity in the first derivative; a transition is of the second order (e.g. glass transition) if the second derivative of the Gibbs free energy exhibits a discontinuity. Volume itself, V, is the first derivative of Gibbs free energy *vs* pressure: $V = \left(\frac{\partial G}{\partial P}\right)_T$, and thus, the derivative of V vs. T is the second (mixed) derivative of the Gibbs free energy: $\left(\frac{\partial V}{\partial T}\right)_P = \left(\frac{\partial^2 G}{\partial T \partial P}\right)$. A discontinuity in $\frac{\partial V}{\partial T}$ is therefore the signature of a second order transition, i.e. the glass transition and this is precisely why kinks in V vs. T are generally used as the strongest experimental evidence of the glass transition of fluids, including water (see for example Figure 1 in Debenedetti & Stillinger, Nature, 410, 259–267 (2001)[h]). As the Reviewer can see, there is an unambiguous discontinuous change of slope in $\frac{\partial V}{\partial T}$ in Figure 4g in samples with 8.3 wt% D_2O and 6.7 wt% H_2O in the reported range (-74 °C to -64 °C); furthermore, this discontinuity is evident both in cooling and heating modes, which is therefore a very strong experimental evidence of Tg. In the revised manuscript on page 9, we have added the adjusted R^2 for the piecewise linear fit model in Figure 4g and removed the Extended Data Figure 6 to avoid misunderstandings. Figure 4g on page 9 of the revised manuscript is modified to:

g

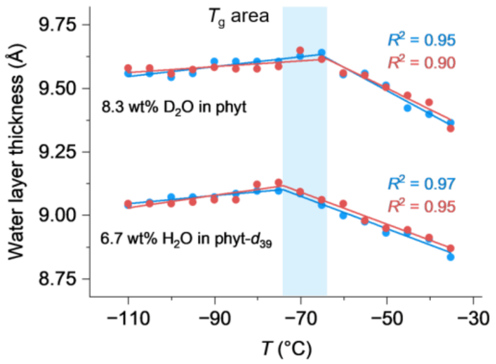

The Figure caption in line 259 of the revised manuscript is modified as follows:

"

***g**, Water layer thickness as a function of temperature in two mesophases, obtained from EDPs as described in Methods. The T_g area is defined by the ~~intersection~~ breakpoints of the ~~fits~~ piecewise linear regression model, applied to the linear regimes of the cooling (blue) and heating (red) cycles. The temperature rate was 1 °C min^{-1}. Curves are offset and rescaled vertically for clarity.*

"

Regarding statistical significance, we did not perform multiple measurements on the same sample. Instead, to demonstrate the consistency of the observed behaviour across samples, we present the temperature dependence of the water layer thickness for two independent samples with different compositions in Figure 4g.

With respect to the comment: "*why does the fluidity of phytantriol imply that temperature dependent changes can only be due to water? Also, liquids have temperature-dependent densities*", we believe that the above lines already respond to this comment: we know from the entire analysis presented in the manuscript that phytantriol is liquid down to at least -105 °C: therefore, in the reported range (-74 °C to -64 °C) discontinuities in $\frac{\partial V}{\partial T}$ cannot arise from phytantriol; in other words, any density change associated to phytantriol cannot exhibit discontinuities above -105 °C, leaving the observed discontinuity solely to the behaviour of nanoconfined water, that is, its glass transition.

h. Debenedetti, P. G. & Stillinger, F. H. Supercooled liquids and the glass transition. *Nature* **410**, 259–267 (2001). https://doi.org/10.1038/35065704

Minor comments

- Line 215, lifetime broadening of vibrational bands is often minor, and I suspect that inhomogeneous broadening and other dephasing mechanisms dominate here. I recommend rephrasing this discussion.

We agree that the observed linewidth is not solely dominated by the intrinsic lifetime of the vibrational mode and thank the Reviewer for this valuable insight. We have clarified this in the revised version of the manuscript in line 223 as follows:

"On the other hand, the width of the stretching mode is inversely proportional to its lifetime[39], although it may include contributions from inhomogeneous broadening mechanisms. The lifetime initially increased monotonically upon cooling (Fig. 4b, orange dots) and, below about −20 °C, increased abruptly before reaching a plateau at −50 °C."

- L223: I suppose there were no deuterium molecules, but D2O molecules.

We thank the Reviewer for pointing out this mistake. We have corrected the sentence to clarify that the ^{2}H ssNMR signal arises from deuterium nuclei in water molecules, rather than from "deuterium molecules". The text in line 231 of the revised manuscript now reads:

"^{2}H ssNMR provides information on the reorientation of water molecules, while the quadrupolar coupling constant (C_Q) reflects the dynamics of the deuterium nuclei within the molecule."

Further editorial changes made by the authors:

- Line 117:

We have removed the article "a" in front of "structural hysteresis":

"By contrast, the mesophase with 15.0 wt% H_2O exhibited ~~a~~ structural hysteresis due to the formation and melting of ice."

- Line 108:

The mixed reverse micellar and lamellar phase was originally denoted as ($L_\alpha + L_2$) in the main text. In the legend and caption of Figure 2c, we denote the mixed phase as ($L_2 + L_\alpha$). We have adapted the main text in line 108 for consistency:

"Upon cooling 7.5 wt% H_2O in phytantriol from 20 to −120 °C, the symmetry shifted from a mixed reverse micellar and lamellar ($L_2 + L_\alpha$) to a highly ordered lamellar phase (L_α), which remained noncrystalline due to the non-freezing properties of phytantriol[29]."

- Lines 136 and 258:

We have deleted "the" in front of "Methods":

*"**c**, Lattice size and phase symmetry (Ia3d, $L_2 + L_\alpha$, L_α) for 7.5 and 15.0 wt% H_2O in phytantriol as a function of temperature during the cooling and heating cycle derived from Bragg reflections as shown in **a** and **b** and described in ~~the~~ Methods."*

*"**g**, Water layer thickness as a function of temperature in two mesophases, obtained from EDPs as described in ~~the~~ Methods."*

- Line 173:

We have replaced the term "full width half maximum" with the more standard form:

"The full-width at half-maximum (FWHM) of the Lorentzian as a function of Q provides information on the time and length scales of the dynamics[32]."

- Lines 174 and 230:

We have used "information on" instead of "information about":

"The full-width at half-maximum (FWHM) of the Lorentzian as a function of Q provides information ~~about~~ on the time and length scales of the dynamics[32]."

"^{2}H ssNMR provides information ~~about~~ on the reorientation of water molecules, while the quadrupolar coupling constant (C_Q) reflects the dynamics of the deuterium nuclei within the molecule."

- Line 187:

We have replaced "high" with "elevated" to improve precision of wording:

"Based on a comprehensive analysis of neutron scattering experiments, we thus identified a remarkably ~~high~~ elevated temperature range for the liquid-to-glass transition of sub-nanometric soft confined water between −35 °C (lower limit determined by QENS analysis) and −21 °C (upper limit given by FWS) on a nanosecond time scale."

- Lines 266 and 477:

We have adapted the term "mean-squared displacement" to be consistent throughout the revised manuscript:

"Specifically, we calculated the mean-squared displacement in the direction parallel to the mid plane of the membrane ($MSD_{//}$) from −3 to −83 °C, reaching simulation times of 1 µs (Fig. 4e)."

"The $MSD_{//}$ is defined as the squared displacement of the position of the oxygen atoms of the water molecules parallel to the membrane mid-plane, averaged over all water molecules:"

- Line 371:

We have revised the sentence in the Methods section to improve precision:

"Backscattering QENS and elastic FWS experiments were performed on the Emu spectrometer with an energy resolution of 1.1 µeV using an incident wavelength of 6.27 Å,…"

- Line 484:

We have added a "Data Availability" section after the Methods section but before the References.

- Lines 630–631:

We have added a funding agent that was previously omitted:

"S.A. acknowledges support from MCIN/AEI/10.13039/501100011033 and FSE+ through a Ramón y Cajal Fellowship (Ref. RYC2022-037744-I), ~~and~~ from MCIN/AEI grant CEX2023-001316-M through the María de Maeztu programme for Units of Excellence in R&D, and from 'Comunidad de Madrid' through the CMOFs4water-CM Project (TEC-2024/ECO-332) within the R&D activities programme 'Tecnologías 2024'."

- We have transferred the previous Extended Data Figures 1–5 to the revised Supplementary Information to comply with the formatting requirements of Nature Communications.

Reviewer #1

We appreciate Reviewers #1's comments on our revised manuscript. Below is our response with references to the lines of the revised manuscript.

The authors have addressed my original comments in a satisfactory way. However, after having submitted my report, I was left with a nagging feeling of unease over the authors' handling of the time-resolution of each experimental technique versus dynamical arrest and the glass transition. The use of a specific length-scale (nearest-neighbor distance) to be traversed within the time-window of an applied experimental technique to distinguish between the liquid and glassy state makes the definition of the glassy state completely arbitrary and can be placed at basically any temperature. One could nowadays in principle go to attosecond resolution or femtosecond and if the molecules didn't move sufficiently during that time-interval define the system as glassy. Applying a different technique, with longer time-window, would then contradict that finding.

We thank the Reviewer for these comments. The identification of the glassy state depends indeed on the time window of the experimental probe (and therefore the length scales involved), and in that sense the transitions discussed here are operational rather than a unique, technique-independent singular temperature. This is precisely why, in our manuscript, we compare methods spanning several orders of magnitude in characteristic times and refrain from assigning a single universal Tg to all observations. In this context, the dependence of the measured transition temperature on the rate/time scale of the measurement is a well-established feature of glass-forming systems, which is very different from 1st order transitions such as melting or evaporation, where transitions are independent (within experimental errors) on timescales probed by different techniques. As a courtesy to the Reviewer, we provide below three well-known examples illustrating the dependence of the glass transition temperature in glassy fluids on:

i) measurement method (expansion coefficient, T_{ga}, vs. isobaric heat capacity, T_{gb}, left panel, Debenedetti & Stillinger, Nature 410, 259–267, 2001);
ii) cooling rate (central panel, Ediger et al. J.Phys.Chem, 100, 13200-13212, 1996);
iii) frequency in specific-heat spectroscopy (right panel, Birge 1986, Phys. Rev. B 34, 1631, 1986).

These examples illustrate well the rate/frequency/technique dependence of the glass transition temperature in glassy fluids.

[REDACTED]

Fig 1. from Debenedetti & Stillinger. 2001

[REDACTED]

Fig 1. from Ediger et al. 1996

[REDACTED]

Fig 1. From Birge 1986

This seems to be the rationale for stating in the abstract that "Remarkably, this range … even extends above the homogeneous nucleation temperature of water." This refers to the range - 63 to -20 C where the measurements were done using micro to picosecond time windows. It is true that -20 C is above the homogeneous nucleation temperature of water, but this is not a relevant comparison. Diffusion is slow at -20 C, but the sample is regular supercooled water. Nothing spectacular about that. Please remove the reference to homogeneous ice nucleation.

Following the Reviewer's advice, we have removed the sentence "*Remarkably, this range … even extends above the homogeneous nucleation temperature of water.*" from line 34 of the abstract of the revised manuscript.

Page 3, lines 79-81. Here, there is a confusing discussion where the authors "refrain to point directly and solely to the T_g" but "more generally refer to liquid-to-glass transition(s)". Typically, T_g refers to the liquid-to-glass transition so it becomes an awkward way of connecting but not quite to T_g.

We thank the Reviewer for pointing this out. Our intention was to avoid implying a single, uniquely defined Tg common to all experimental probes used in this work. Specifically, the glass transition refers to the α-relaxation, i.e. the dynamic arrest over length-scales comparable to the individual constitutive molecule (at the time-scaled probed); whereas the β-relaxation is faster than α-relaxation because it occurs at lower length-scales. Given the very vast range of time scales probed in our work, it is possible that both α- and β-relaxations are indeed probed (especially down to 10^{-12} s timescales). For this reason, we used the terminology "liquid-to-glass transition(s)" with an "s" at the end to remain broader than single α-relaxation. For the sake of clarity, we have decided to substitute liquid-to-glass transition(s)" with "dynamic arrest on the experimental time scale" in line 79 of the revised manuscript as follows:

"However, given the extremely vast range of time scales probed here by a multitude of diverse methods, we refrain to point directly and solely to the T_g, and we more generally refer to ~~liquid-to-glass transition(s)~~ dynamic arrest on the experimental time scale, accounting for possible observations of both α- and β-relaxations."

Page 3, lines 82-83. The authors need to define what they mean by "dynamic arrest" as it is clearly related to the time-scale of each measurement and not a general property of the system.

We agree with the Reviewer here and we have modified the previous lines 82-83 to:

"We then reach a strikingly coherent picture in which dynamic arrest, that is, the marked suppression of relaxation over the time window probed by the experimental techniques employed here, occurs in a temperature range significantly higher than previously reported for soft nanoconfined water."

Page 9, lines 258 and 261. Here, the authors refer directly to "the T_g areas" and "the T_g range of water in…". What the data really show is the slowing down of thermal motion to make said motion undetectable by specific techniques, but it doesn't indicate what is usually meant by a glassy state.

We thank the Reviewer for this comment, and we have made the following changes:

a) At line 258, the term "Tg area" has been changed to "Tg range". As in this instance we use the classic Tg volumetric interpretation of glass transition - see Figure 1 in Debenedetti, P. G. & Stillinger, F. H.

Supercooled liquids and the glass transition. *Nature* 410, 259–267 (2001). https://doi.org/10.1038/35065704 - the use of "Tg" is appropriate and justified here.

b) Line 261. The Reviewer is totally correct here, precisely for the arguments discussed above. We have therefore revised the sentence at line 261 into:
"Overview *of the dynamic arrest temperature range* (in blue) determined in this work by various techniques". We wish to thank the Reviewer for having captured this.

Page 11, lines 302-303. "Our results indicate that, as expected, the exact location of the liquid-to-glass transition depends on the dynamical time scale probed by the measurement technique." This will be true of any definition in terms of a length-scale traversed during the experimental time-window. Improving the time-resolution will move the so-defined T_g upwards in temperature, while the result of other, slower techniques, would remain the same. I would suggest the authors define exactly what they mean by liquid-to-glass transition since they clearly refer to measurement techniques rather than what is typically (and intuitively) meant by a glassy state.

We are grateful to the Reviewer for prompting us to sharpen the terminology. We believe that the revised wording now clarifies the distinction between a single universal Tg and the broader temperature interval over which different probes detect dynamical arrest, while preserving the central conclusion of the manuscript: under soft nanoconfinement, water exhibits a broad and internally consistent crossover from liquid-like to arrested dynamics across multiple experimental time scales.

I see the present contribution as very valuable and, in particular, the identification of a region where part of the system is glassy while the other remains liquid is quite intriguing. However, I urge the authors to reconsider (see above) their handling of the connection to T_g. A definition based, e.g., on the longest available time-scale (rather than an arbitrary one) or thermodynamical response when going from glass to liquid seems less arbitrary to the present reviewer.

Still, being able to determine at which temperature the "maximum velocity" slows down enough to not reach a given distance on a given time-scale is definitely of value.

We are thankful to the Reviewer for these additional comments and for allowing us to further polish the terminology and the language: we are confident that the additional changes have further improved the storyline of the submitted revised manuscript.

Reviewer #2

We appreciate Reviewers #2's comments on our revised manuscript. Below is our response with references to the lines of the revised manuscript.

The authors have convincingly revised their manuscript in response to my previous comments. In my view, the manuscript is now acceptable for publication in Nature Communications subsequent to very minor additional revisions addressing the items listed below:

The 90% transmission of the samples for neutron spectroscopy given in the Methods section appears to be a rough estimate: "The samples were loaded in annular cans with 0.2 mm gap size ensuring a 90% transmission." It should either be clearly stated that it is an estimate, or the transmission should be calculated.

We thank the Reviewer for pointing this out. The Reviewer is right in that the reported transmission of 90% represents an estimate based on the sample geometry and composition, as well as practical limitations in achieving perfectly homogeneous filling of viscous samples in the annular sample cans. We have clarified this in the revised manuscript by explicitly stating that the value is estimated. The modified sentence in line 377 of the revised manuscript now reads:

"The samples were loaded in annular cans with 0.2 mm gap size, corresponding to an estimated transmission of approximately 90% ~~ensuring 90% transmission~~."

It may be helpful to quote the temperature protocol from the Methods section in the caption of the new supplementary figure 7, i.e., the fact that the data were recorded upon heating from approximately 4K. This heating ramp rules out any simple freezing-point offset of bulk water as an alternative explanation of the transition seen at approximately -25 deg. C in the MSD, which is, thus, rightly attributed to the Tg of confined water in this system.

We thank the Reviewer for this comment and have modified the caption of supplementary Fig. 7 on page 5 in the revised supplementary information as follows:

*"**Supplementary Fig. 7.** Apparent mean-squared displacement $\langle u^2 \rangle$ vs. temperature of 6.7 wt% H_2O in phytantriol-d_{39} derived from the elastic FWS data shown in Fig. 3g. The T_g of water is evident at around −25 °C by a sudden change in the slope of the mean-squared displacement. Data were recorded during a temperature ramp from −269 to 27 °C at a rate of 0.5 °C min^{-1}, ensuring near-equilibrium conditions and minimising thermal lag. The Q-range considered for the analysis was 0.32–0.92 $Å^{-1}$."*

Regarding the new statement in the Methods section, "for QENS analysis of the backscattering experiments, the lowest practical Q-value used in the analysis was 0.42 Å−1, owing to insufficient counting statistics in the lowest-Q bin at the measured temperatures.": Could it be that at the lowest Q, the energy resolution is also insufficient, since the lowest Q detector might be slightly out of exact backscattering, and therefore have a broader resolution than the higher Q detectors? Also, the effective analyzer surface area seen at lowest Q might be too small, explaining the low signal.

We thank the Reviewer for this comment. Measurements at low *Q* are generally more challenging, both in terms of counting statistics and geometry of the spectrometer. However, the lowest-*Q* detector is no more offset from exact backscattering than the others and therefore does not have an intrinsically broader energy resolution. The observed reduction in signal at low *Q* arises from a characteristic specific to the Emu backscattering spectrometer. For operational safety reasons, a forward beam stop is required to capture incident neutrons that are not reflected onto the Doppler drive. Mounting this beam stop partially shades the lowest-*Q* analyser/detector, which effectively reduces the accessible analyser surface area and thus the detected intensity.

Reviewer #3

The authors have addressed all my comments appropriately. I recommend publication of the manuscript in it present form.

We thank Reviewer #3 for the positive evaluation and are pleased that our revisions have addressed their concerns.

Report on manuscript NCOMMS-25-91466-T

This is an interesting study combining a significant number of measurement techniques with results from careful simulations to elucidate the process of dynamic arrest in water in soft confinement. This provides interesting insight into a regime intermediate between bulk liquid and water confined in fixed nanometer size pores. It is of particular interest to see the diverse ranges in temperature as determined using different techniques, probing the dynamics on different time-scales. Maybe not surprising, but still interesting was to see the intermediate regime with glassy water confined between still liquid tails. The presentation is quite clear and I have very few comments on some aspects where the authors could add some clarification. Otherwise than that, I feel the manuscript is publishable after the minor comments below have been addressed.

1) Line 47. Glassy water in the form of the amorphous LDA and HDA forms do exhibit structural order, but only at short range. Please consider adding "long-range" to describe the structural order of the crystalline phases.

2) Line 112. It is unclear what "supercoolong effects" are referred to. Please specify since effects of supercooling can manifest in many different properties.

3) Lines 149-150. The measurements show an enhanced mobility of H_2O over D_2O, but the jump to "ergodically" exploring the phase space seems rather large since this would (formally) require proof that "all" possible configurations are explored on the relevant time scale.

4) Line 289. The "rate-dependence" needs to be specified. The rate of change in temperature seems to have been constant. I assume the authors mean the time-scale of the measurements, but it would be good to clarify.

Report on manuscript NCOMMS-25-91466A

The authors have addressed my original comments in a satisfactory way. However, after having submitted my report, I was left with a nagging feeling of unease over the authors' handling of the time-resolution of each experimental technique versus dynamical arrest and the glass transition. The use of a specific length-scale (nearest-neighbor distance) to be traversed within the time-window of an applied experimental technique to distinguish between the liquid and glassy state makes the definition of the glassy state completely arbitrary and can be placed at basically any temperature. One could nowadays in principle go to attosecond resolution or femtosecond and if the molecules didn't move sufficiently during that time-interval define the system as glassy. Applying a different technique, with longer time-window, would then contradict that finding.

This seems to be the rationale for stating in the abstract that "Remarkably, this range … even extends above the homogeneous nucleation temperature of water." This refers to the range -63 to -20 C where the measurements were done using micro to picosecond time windows. It is true that -20 C is above the homogeneous nucleation temperature of water, but this is not a relevant comparison. Diffusion is slow at -20 C, but the sample is regular supercooled water. Nothing spectacular about that. Please remove the reference to homogeneous ice nucleation.

Page 3, lines 79-81. Here, there is a confusing discussion where the authors "refrain to point directly and solely to the T_g" but "more generally refer to liquid-to-glass transition(s)". Typically, T_g refers to the liquid-to-glass transition so it becomes an awkward way of connecting but not quite to T_g.

Page 3, lines 82-83. The authors need to define what they mean by "dynamic arrest" as it is clearly related to the time-scale of each measurement and not a general property of the system.

Page 9, lines 258 and 261. Here, the authors refer directly to "the T_g areas" and "the T_g range of water in…". What the data really show is the slowing down of thermal motion to make said motion undetectable by specific techniques, but it doesn't indicate what is usually meant by a glassy state.

Page 11, lines 302-303. "Our results indicate that, as expected, the exact location of the liquid-to-glass transition depends on the dynamical time scale probed by the measurement technique." This will be true of any definition in terms of a length-scale traversed during the experimental time-window. Improving the time-resolution will move the so-defined T_g upwards in temperature, while the result of other, slower techniques, would remain the same. I would suggest the authors define exactly what they mean by liquid-to-glass transition since they clearly refer to measurement techniques rather than what is typically (and intuitively) meant by a glassy state.

I see the present contribution as very valuable and, in particular, the identification of a region where part of the system is glassy while the other remains liquid is quite intriguing. However, I urge the authors to reconsider (see above) their handling of the connection to T_g. A definition based, e.g., on the longest available time-scale (rather than an arbitrary one) or thermodynamical response when going from glass to liquid seems less arbitrary to the present reviewer.

Still, being able to determine at which temperature the "maximum velocity" slows down enough to not reach a given distance on a given time-scale is definitely of value.
